# Unveiling Novel Drug Targets and Emerging Therapies for Rheumatoid Arthritis: A Comprehensive Review

**DOI:** 10.1021/acsptsci.4c00067

**Published:** 2024-05-28

**Authors:** Manoj Khokhar, Sangita Dey, Sojit Tomo, Mariusz Jaremko, Abdul-Hamid Emwas, Rajan Kumar Pandey

**Affiliations:** † Department of Biochemistry, All India Institute of Medical Sciences, Jodhpur, 342005 Rajasthan, India; ‡ CSO Department, Cellworks Research India Pvt Ltd, Bengaluru, 560066 Karnataka, India; § Smart-Health Initiative (SHI) and Red Sea Research Center (RSRC), Division of Biological and Environmental Sciences and Engineering (BESE), King Abdullah University of Science and Technology (KAUST), Thuwal, 23955 Jeddah, Saudi Arabia; ∥ Core Laboratories, King Abdullah University of Science and Technology (KAUST), Thuwal 23955-6900, Kingdom of Saudi Arabia; ⊥ Department of Medical Biochemistry and Biophysics, Karolinska Institute, Stockholm 17177, Sweden

**Keywords:** rheumatoid arthritis, joints, bone, therapeutics, biologicals, drugs, JAKs, noncoding RNAs

## Abstract

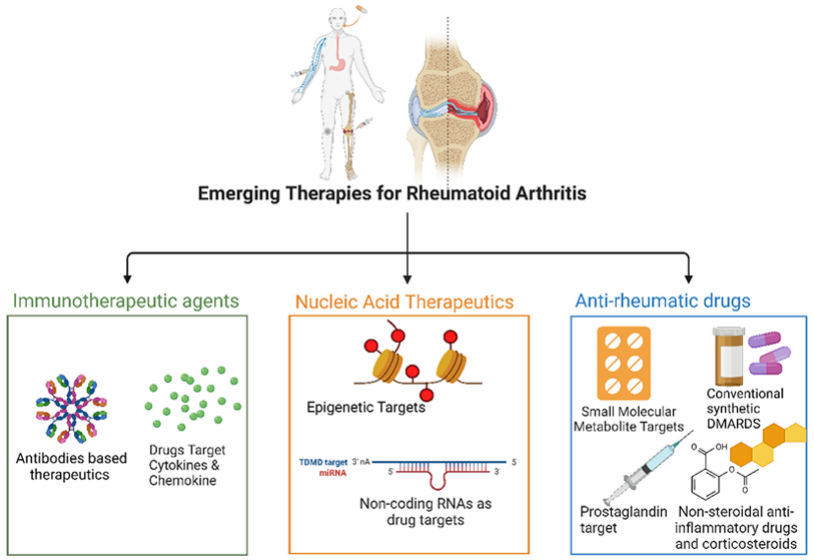

Rheumatoid arthritis (RA) is a chronic debilitating autoimmune disease, that causes joint damage, deformities, and decreased functionality. In addition, RA can also impact organs like the skin, lungs, eyes, and blood vessels. This autoimmune condition arises when the immune system erroneously targets the joint synovial membrane, resulting in synovitis, pannus formation, and cartilage damage. RA treatment is often holistic, integrating medication, physical therapy, and lifestyle modifications. Its main objective is to achieve remission or low disease activity by utilizing a “treat-to-target” approach that optimizes drug usage and dose adjustments based on clinical response and disease activity markers. The primary RA treatment uses disease-modifying antirheumatic drugs (DMARDs) that help to interrupt the inflammatory process. When there is an inadequate response, a combination of biologicals and DMARDs is recommended. Biological therapies target inflammatory pathways and have shown promising results in managing RA symptoms. Close monitoring for adverse effects and disease progression is critical to ensure optimal treatment outcomes. A deeper understanding of the pathways and mechanisms will allow new treatment strategies that minimize adverse effects and maintain quality of life. This review discusses the potential targets that can be used for designing and implementing precision medicine in RA treatment, spotlighting the latest breakthroughs in biologics, JAK inhibitors, IL-6 receptor antagonists, TNF blockers, and disease-modifying noncoding RNAs.

Rheumatoid arthritis (RA) is a chronic autoimmune disease, that causes inflammation, deformities, and loss of joint function. It can also impact organs like the skin, eyes, lungs, and blood vessels.^1^ It is characterized by aggressive synovial hyperplasia, synovial membrane hypertrophy, and bone erosion (Figure 1). Multiple factors, including genetics, environment, lifestyle, obesity, autophagy, and immune system dysregulation, contribute to disease development.^2^ Interestingly, although having a familial history of RA can increase a person’s risk of developing the condition 3-fold, many RA patients do not have a familial history.^3^ Cigarette smoking is the primary environmental factor that establishes seropositive RA.^4^ Smoking induces stress on lung tissues, leading to the peptidyl arginine deiminase (PAD4)-mediated conversion of arginine to citrulline. This affects various intracellular proteins (histone) and matrix proteins (collagen, vimentin, enolase, fibronectin, and fibrinogen).^5^ Other factors contributing to RA susceptibility include increased body mass index (BMI), inflammatory dietary patterns, and dental problems.^6−9^ RA primarily impacts aged people between 50 and 60 years old, with a global prevalence of around 0.5% among adults. Women are affected 2–3 times more frequently than men.^10^

**Figure 1:**
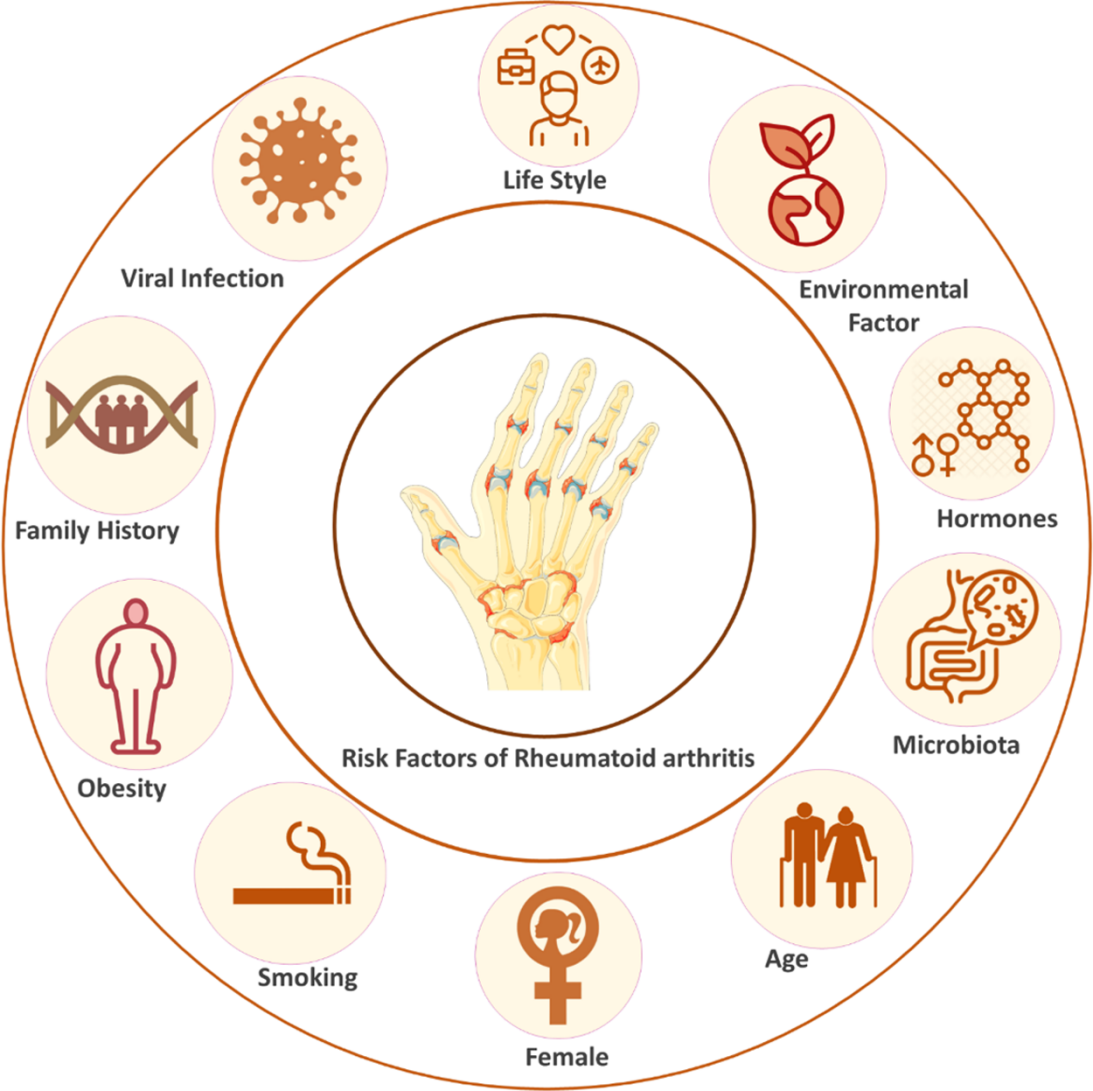
Risk factors for RA are lifestyle, environment, hormones, microbiota, age, sex, smoking status, obesity, family history and viral infection (Created using Biorender).

This comprehensive review presents the contemporary landscape of molecular therapeutic targets and strategies for managing RA. Moreover, it highlights the potential advantages of multitarget therapy and precision medicine in RA treatment, spotlighting the latest breakthroughs in biologics, JAK inhibitors, IL-6 receptor antagonists, and TNF blockers.

## 1 Deciphering Persistent Pain in Rheumatoid Arthritis

Persistent pain is a significant issue for patients with RA, even in remission.^11^ The pain, often multifactorial, can be due to active inflammation, joint damage, or central pain regulation alterations.^12^ The management of RA should prioritize the alleviation of pain to improve patients’ quality of life. This is a crucial aspect of RA management, as the disease can lead to significant disability and mortality if left untreated.^13^ Patients with RA often prioritize autonomy, independence, and overall health. Persistent pain is a major concern for them.^14^ Comprehensive evaluation and specific interventions tailored to individual patients’ pain and circumstances are also important in pain management for rheumatic diseases.^15^ Chronic pain in RA involves multiple mechanisms. In addition to joint inflammation, peripheral sensitization, and central nervous system abnormalities play a significant role in RA-associated pain.^16^ The long-term prognosis for pain is often unfavorable, even after inflammation is suppressed, and pain is associated with fatigue and psychological distress. The role of cytokines, such as TNF-α, IL-1, and IL-6, in the pathogenesis and associated pain has been well characterized.^17^

## 2 The Immunological Events of Rheumatoid Arthritis

**Figure 2:**
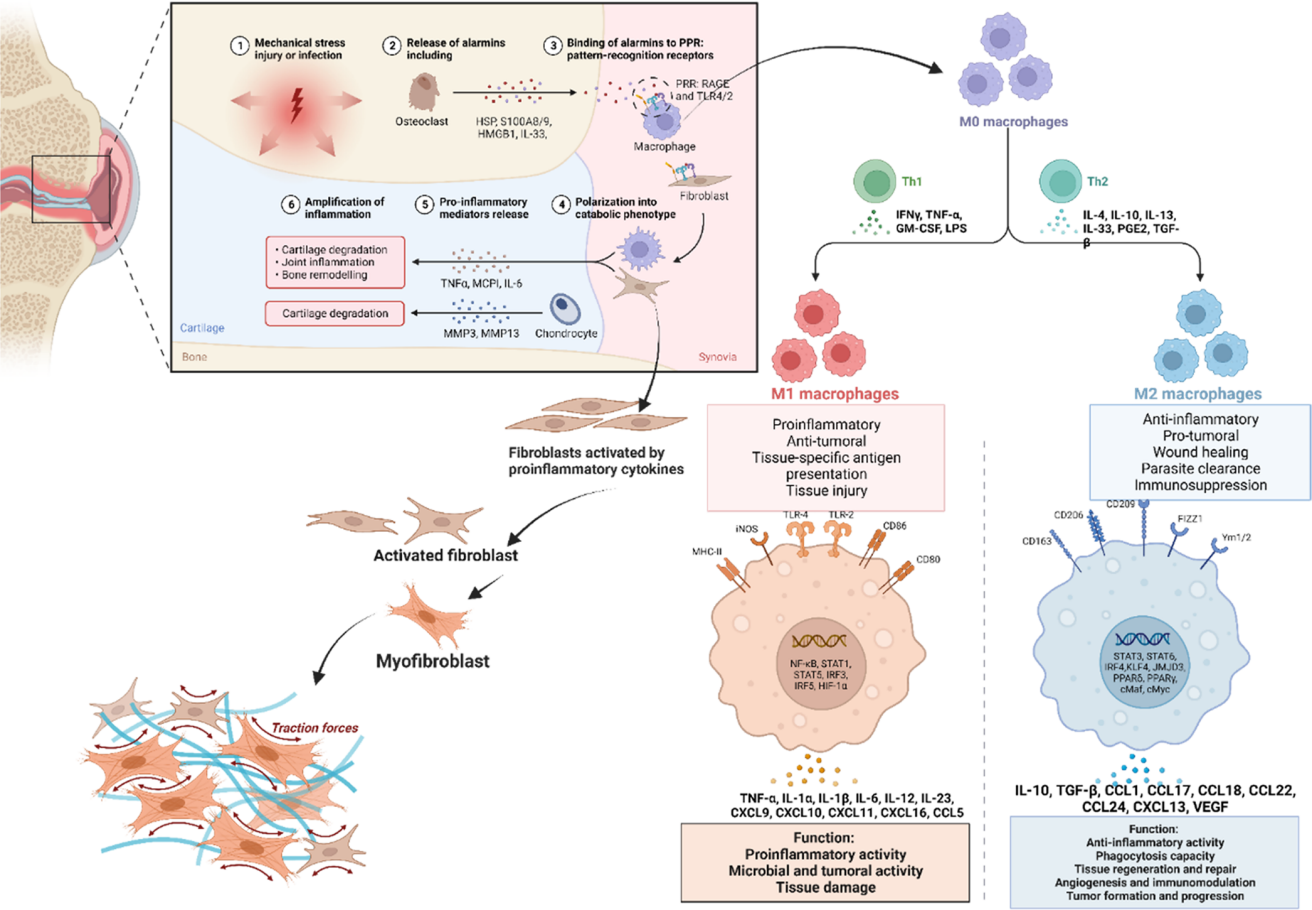
Immune regulatory role of macrophages in rheumatoid arthritis (RA). M1 macrophages release pro-inflammatory cytokines, causing joint inflammation and tissue damage. M2 macrophages secrete anti-inflammatory cytokines, promoting tissue repair and resolving inflammation. The balance between M1 and M2 macrophages shifts during RA progression, with an increase in M2 macrophages over time. The diagram also illustrates synovial fibroblasts (SFs) interacting with immune cells and the extracellular matrix (ECM) in the RA joint microenvironment, contributing to inflammation through cytokine production and matrix degradation (Created using Biorender).

**Figure 3:**
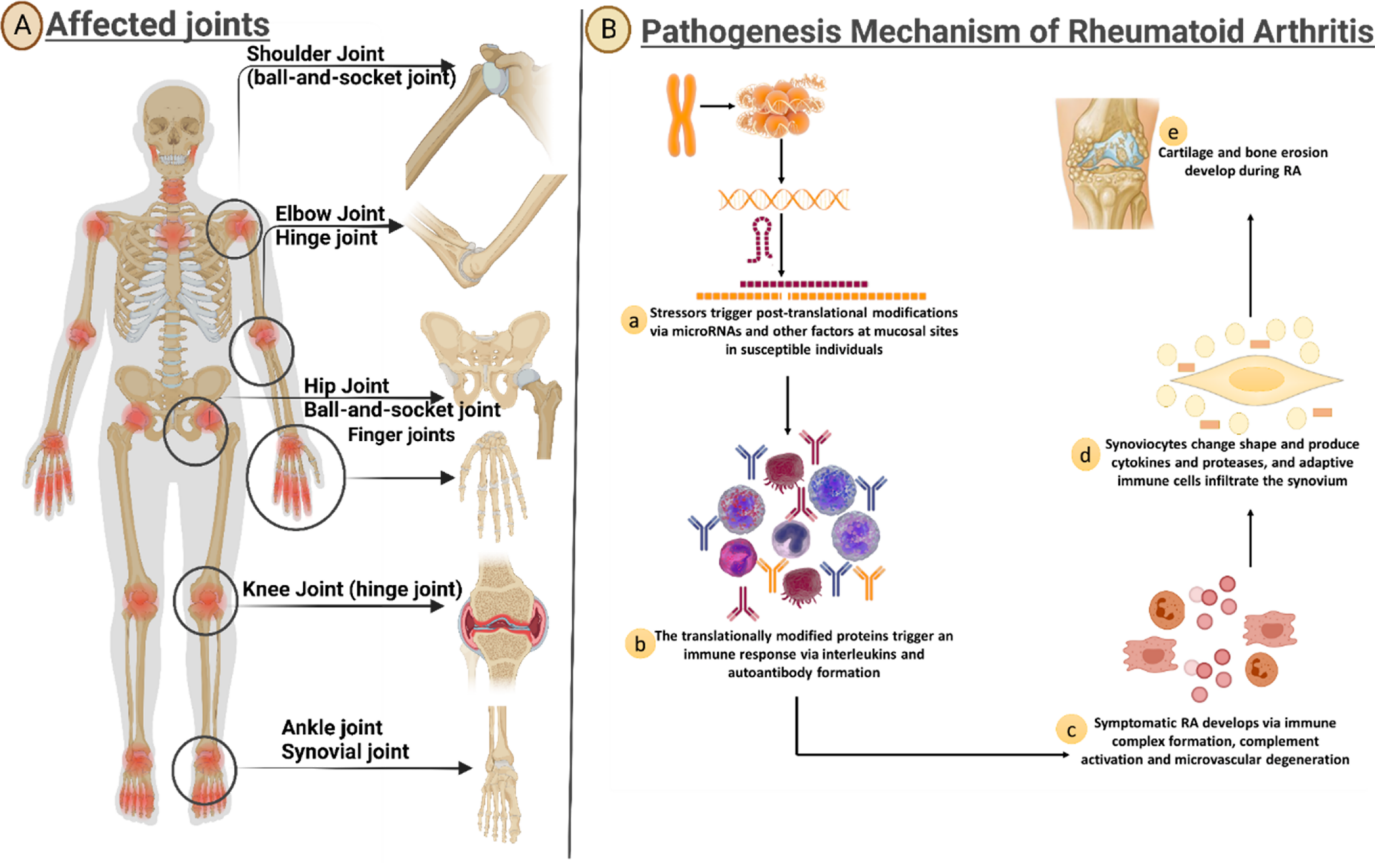
(A) RA commonly affects small joints in the fingers, hands, wrists, knees, elbows, shoulders, and feet. (B) Pathogenesis of RA progression: (a) Stressors trigger post-translational modifications via interference with noncoding RNAs at the mucosal site in RA patients. (b) Translational modifications initiate an immune response through interleukins, chemokines, transcription factors, and the production of autoantibodies. (c) Immune complex formation, complement activation, or microvascular degeneration can result in symptomatic RA. (d) Changes in synoviocytes induce cytokine and protease production, leading to infiltration of adaptive immune cells into the synovium. (e) Synovial expansion causes inflammation, cartilage damage, bone erosion, and the development of RA (Created using Biorender).

The pathogenesis of RA involves various immune cells and cytokines, which are essential in the development of disease.^18^ Healthy synovium consists of fibroblast-like synoviocytes (FLS) and macrophage-like synoviocytes (MLS).^19^ The pathogenic synovium exhibits thickened intimal lining and activated FLS and MLS. FLS produces matrix metalloproteinases (MMPs), IL-6, leukotrienes, and prostaglandins, while MLS produces TNF, IL-1, and IL-6, all contributing to RA symptoms.^20,21^ Infiltrating CD4^+^ memory T cells and Th1 cells create a pro-inflammatory environment, with TNF-α, IFN-γ, and IL-1β causing cartilage and bone damage.^22,23^ Ectopic germinal canters may form in some patients, producing antibodies like ACPAs and RF, leading to RA development.^24^ Synovial invasion into adjacent tissue causes cartilage and bone damage (Figure 2). HLA-DRB1 and PTPN22 are genetic factors associated with RA pathogenesis, influencing adaptive immunity and increasing disease risk.^25,26^ The 1858T variant of PTPN22, along with ACPAs, is a strong predictor of disease development. HLA-DRB1 encodes the MHC-II protein, while PTPN22 encodes the cytoplasmic lymphoid specific phosphatase (LYP), regulating T and B-cell activation^27^ (Figure 3).

## 3 Current Therapeutic Developments for Rheumatoid Arthritis

**Figure 4:**
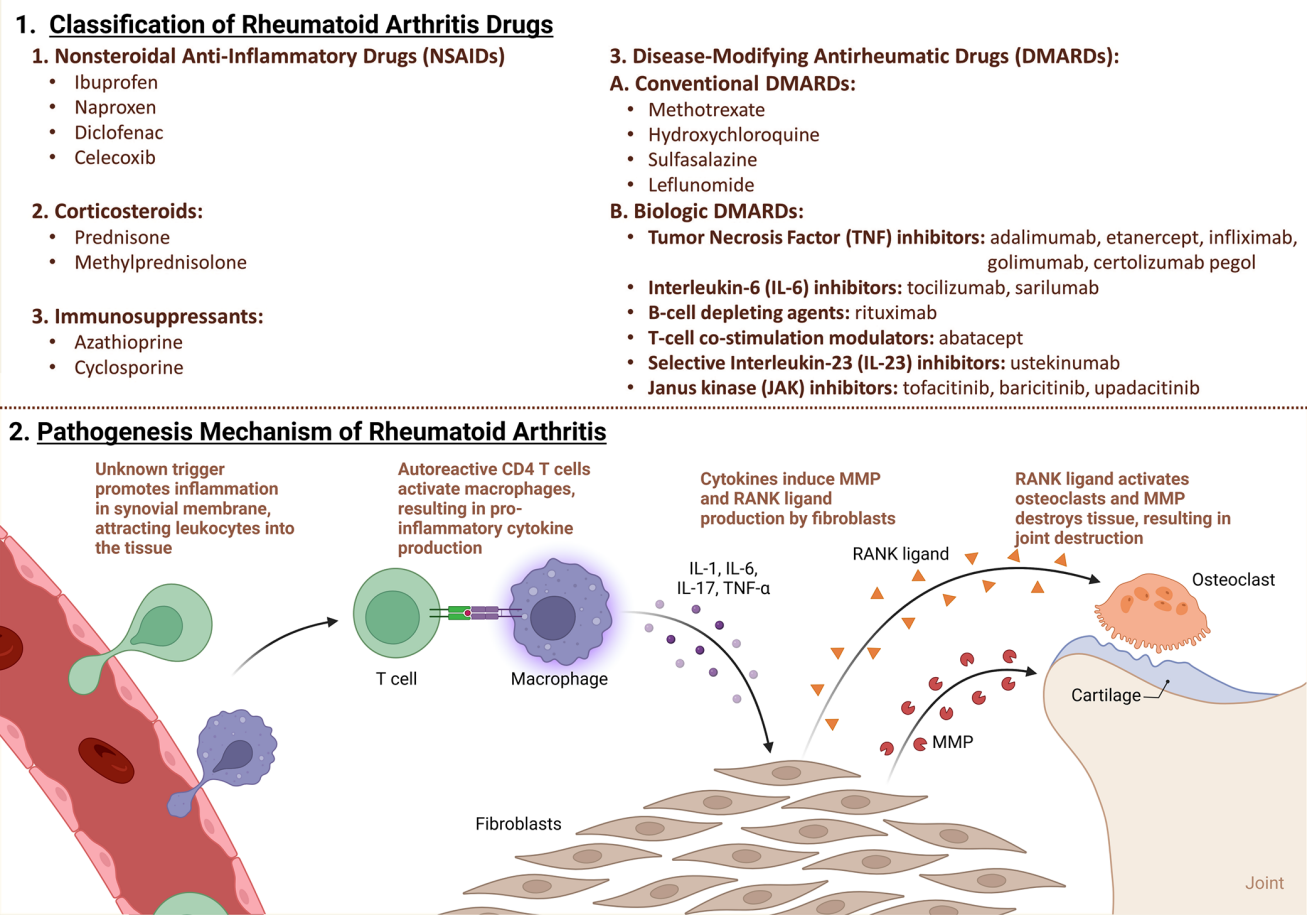
(1) RA drug classification and pathogenesis: Drug classes for RA management include NSAIDs for symptomatic relief, corticosteroids to suppress inflammation, and DMARDs (conventional and biologic) targeting specific immune components. Biologic DMARDs encompass TNF inhibitors, IL-6 inhibitors, B-cell depleting agents, T-cell costimulation modulators, selective IL-23 inhibitors, and JAK inhibitors. Immunosuppressants like azathioprine and cyclosporine may also be used. (2) RA pathogenesis involves immune factors. Activated immune cells release pro-inflammatory cytokines, causing synovial inflammation and joint damage. Interaction with bone and cartilage exacerbates the damage. Autoantibodies like RF and anti-CCP antibodies, used for diagnosis, are produced due to cytokine stimulation. Genetic and environmental factors, such as smoking and infections, trigger immune cell activation (Created using Biorender).

Safe and effective therapeutic approaches are crucial for regulating inflammation and reducing disease activity in RA. This can be achieved using DMARDs, which can be traditional or biological.^28^ Synthetic DMARDs like methotrexate, hydroxychloroquine, sulfasalazine, tofacitinib, and leflunomide suppress the immune system and slow disease progression.^10^ Methotrexate is often the first-line therapy, while hydroxychloroquine can be used as initial monotherapy for mild symptoms.^10,29^ Sulfasalazine is an alternative for methotrexate intolerance.^30,31^ Tofacitinib and leflunomide are effective for moderate to severe RA. However, conventional DMARDs can have toxic effects such as liver toxicity, teratogenicity, retinal toxicity, gastrointestinal intolerance, renal toxicity, and headache^32^ (Figure 4). Biologic DMARDs, such as infliximab, adalimumab, etanercept, certolizumab pegol, golimumab, rituximab, abatacept, sarilumab, anakinra, and tocilizumab, target key inflammatory pathways and are used when traditional DMARDs show inadequate responses^33,34^ (Table 1). Infliximab neutralizes TNF-α, anakinra, and tocilizumab targets IL-1β and IL-6, respectively, while rituximab and abatacept target B cells and T cells. Biologic DMARDs, being antibodies, impact the immune response and increase susceptibility to infection. Nonsteroidal anti-inflammatory drugs (NSAIDs) and glucocorticoids are additional treatment options. NSAIDs alleviate pain and inflammation by blocking cyclooxygenase (COX-1 and COX-2) enzymes.^35^ By blocking their production, NSAIDs provide quick pain relief for RA patients.^36^ Glucocorticoids, like prednisone and cortisone, are steroid hormones with potent anti-inflammatory and immunosuppressive properties.^37^ They can be used as a bridge therapy for RA patients to rapidly reduce pain and stiffness during high disease activity.^38,39^ While DMARDs can be more effective in managing RA over the long-term, they may take weeks to months to show their full effect^40^ (Table 2).

**Table 1:** Biologicals Used for Treating RA and Their Target Proteins^a^

**Agent**	**Class of target protein**	**Specific target**	**Status**	**References**
Adalimumab	Cytokines	TNF	Marketed	(236)
Anakinra	Cytokines	IL-1R	Marketed	(236)
Canakinumab	Cytokines	IL-1	Marketed	(236)
Certolizumab	Cytokines	TNF	Marketed	(236)
Clazakizumab	Cytokines	IL-6a	Marketed	(236)
Etanercept	Cytokines	TNF	Marketed	(236)
Gevokizumab	Cytokines	IL-1	Marketed	(236)
Golimumab	Cytokines	TNF	Marketed	(236)
Infliximab	Cytokines	TNF	Marketed	(236)
Olokizumab	Cytokines	IL-6a	Marketed	(236)
Sarilumab	Cytokines	IL-6a	Marketed	(236)
Sirukumab	Cytokines	IL-6a	Marketed	(236)
Tocilizumab	Cytokines	IL-6R	Marketed	(236)
BX147	Chemokines	CCR1	Animal study	(237)
Plerixafor	Chemokines	CXCR4	Animal study	(238)
AMG-714	Cytokines	IL-15	Phase 2	(239)
Repertaxin	Chemokines	CXCR1/2	Animal study	(240)
Met-RANTES	Chemokines	CCR5	Phase 2	(241)
IgG1 12–81	Chemokines	CXCL16	Animal study	(242)
ABN912	Chemokines	CCL2	Phase 1	(243)
MLN1202	Chemokines	CCR2	Phase 2a	(244)
AMD3100	Chemokines	CXCR4	Animal study	(245)
CCX733	Chemokines	CXCR7	Animal study	(245)
AZD5672	Chemokines	CCR5	Phase 2	(246)
Rituximab	Cellular Proteins	CD20	Phase 3	(247)
MDX–1100	Chemokines	CXCL10	Phase 2	(248)
Secukinumab	Cytokines	IL-17	Phase 3	(248)
STA 5326 mesylate	Cellular Proteins	Il-23	Phase 2	(249)
rhIL-18BP	Cytokines	IL-18	Phase 1	(250)
Gimsilumab	Cellular Proteins	GM-CSF	Phase 1	(251)
Mavrilimumab	Cellular Proteins	GM-CSF	Phase 2	(251)
Namilumab	Cellular Proteins	GM-CSF	Phase 2	(251)
Otilimab	Cellular Proteins	GM-CSF	Phase 3	(251)
CC-292	Cellular Proteins	BTK	Phase 2	(252)
Andecaliximab	Cellular Proteins	MMP-9	Phase 2	(253)
mAb470	Chemokines	CXCL13	Animal study	(254)
AMG487	Chemokines	CXCR3	Animal study	(255)
JN-2	Chemokines	CXCR3	Animal study	(256)
T140	Chemokines	CXCR4	Animal study	(257)
J–113863	Chemokines	CCR1	Animal study	(258)
8H3–16A12	Chemokines	CCR7	Animal study	(259)
DF2162	Chemokines	CXCR1/2	Animal study	(260)
Ixekizumab	Cytokines	IL-17	Phase 2	(261)
BAY1830839	Cellular Proteins	IRAK-4	Phase 1	(262)
BAY1834845	Cellular Proteins	IRAK-4	Phase 1	(262)
CA-4948	Cellular Proteins	IRAK-4	Phase 2	(262)
PF-06650833	Cellular Proteins	IRAK-4	Phase 2	(262)
E6011	Chemokines	CX3CL1	Phase 1	(263)
Abatacept	Cellular Proteins	CD80	Marketed	(264)
Dekavil	Cytokines	IL-10	Phase 1	(265)
CCX354	Chemokines	CCR1	Phase 2	(266)
SCH351125	Chemokines	CCR5	Phase 1b	(267)
SCH546738	Chemokines	CXCR3	Animal study	(268)
CCX8037	Chemokines	CCR9	Animal study	(269)
Maraviroc	Chemokines	CCR5	Terminated	(270)
SCH–X82	Chemokines	CCR5	Phase 2	(237)
Baricitinib	Cellular Proteins	JAK	Approved	(271)
Tofacitinib	Cellular Proteins	JAK	Approved	(271)
Upadacitinib	Cellular Proteins	JAK	Approved	(271)
Peficitinib	Cellular Proteins	JAK	Phase 3	(271)
Otelixizumab	Cellular Proteins	CD3	Phase 1	(272)
30D8	Chemokines	CXCL12	Animal study	(273)
p8A MCP-1	Chemokines	CCL2	Animal study	(274)
ARRY-371797	Cellular Proteins	p38 MAPK	Phase 1	Clinicaltrials.gov
GS-4059	Cellular Proteins	BTK	Phase 1	Clinicaltrials.gov
HM71224	Cellular Proteins	BTK	Phase 1	Clinicaltrials.gov
ICP-022	Cellular Proteins	BTK	Phase 1	Clinicaltrials.gov
MDX-1342	Cellular Proteins	CD19	Phase 1	Clinicaltrials.gov
ARRY-162	Cellular Proteins	MEK	Phase 2	Clinicaltrials.gov
BAY86–5047	Chemokines	CCR1	Phase 2	Clinicaltrials.gov
BMS-582949	Cellular Proteins	p38 MAPK	Phase 2	Clinicaltrials.gov
BMS-817399	Chemokines	CCR1	Phase 2	Clinicaltrials.gov
Efalizumab	Cellular Proteins	CD11a	Phase 2	Clinicaltrials.gov
INCB018424	Cellular Proteins	JAK	Phase 2	Clinicaltrials.gov
Itacitinib	Cellular Proteins	JAK	Phase 2	Clinicaltrials.gov
M2951	Cellular Proteins	BTK	Phase 2	Clinicaltrials.gov
MK–0812	Chemokines	CCR2	Phase 2	Clinicaltrials.gov
NI-0101	Cellular Proteins	TLR4	Phase 2	Clinicaltrials.gov
Paroxetine	Cellular Proteins	GRK2	Phase 2	Clinicaltrials.gov
PH-797804	Cellular Proteins	p38 MAPK	Phase 2	Clinicaltrials.gov
RO4402257	Cellular Proteins	p38 MAPK	Phase 2	Clinicaltrials.gov
Ruxolitinib	Cellular Proteins	JAK	Phase 2	Clinicaltrials.gov
SB-681323	Cellular Proteins	p38 MAPK	Phase 2	Clinicaltrials.gov
SCIO-469	Cellular Proteins	p38 MAPK	Phase 2	Clinicaltrials.gov
Tasocitinib	Cellular Proteins	JAK	Phase 2	Clinicaltrials.gov
VX-702	Cellular Proteins	p38 MAPK	Phase 2	Clinicaltrials.gov
ZK811752	Chemokines	CCR1	Phase 2	Clinicaltrials.gov
Filgotinib	Cellular Proteins	JAK	Phase 3	Clinicaltrials.gov
Ofatumumab	Cellular Proteins	CD20	Phase 3	Clinicaltrials.gov
VX-509	Cellular Proteins	JAK	Phase 3	Clinicaltrials.gov
Brodalumab	Cytokines	IL-17R	Terminated	Clinicaltrials.gov
Fontolizumab	Cytokines	IFN-g	Terminated	Clinicaltrials.gov
Guselkumab	Cellular Proteins	Il-23	Terminated	Clinicaltrials.gov
Lenzilumab	Cellular Proteins	GM-CSF	Terminated	Clinicaltrials.gov
MEDI5117	Cytokines	Il-2	Terminated	Clinicaltrials.gov
Ocrelizumab	Cellular Proteins	CD20	Terminated	Clinicaltrials.gov
Rilonacept	Cytokines	IL-1	Terminated	Clinicaltrials.gov
MC–21	Chemokines	CCR2	Animal study	(275)

a The target proteins of these biologicals are essential in the development and progression of the disease. These biologicals are designed to target specific proteins, thereby reducing inflammation and slowing down the progression of the disease.

**Table 2:** Small Molecules Used as Drugs in RA and Their Binding Proteins

**Agent**	**Class of Target**	**Target**	**Status**	**References**
Cinnamaldehyde	ROS targets	ROS	*In vitro*	(276)
Eugenol	ROS targets	ROS	*In vitro*	(276)
MK0524	PGs targets	PGD2	*In vivo*	(277)
ER-819762	PGs targets	PGE2	*In vivo*	(278)
CR6086	PGs targets	PGE2	*In vivo*	(279)
15d-PGJ2	PGs targets	PGJ2	*In vivo*	(280)
AL-8810	PGs targets	PGF2a	*In vivo*	(281)
SQ29548	PGs targets	TXA2	*In vivo*	(282)
Montelukast	LTs targets	CysLT1R	*In vivo*	(283)
BML-111	LXs targets	ALX	*In vivo*	(284)
WB2086	PAF targets	PAFR	*In vivo*	(285)
L-NAME	NO targets	iNOS	*In vivo*	(286)
HU-308	Small molecular targets	CB2	*In vivo*	(287)
JWH-015	Small molecular targets	NA	*In vivo*	(288)
URB597	Small molecular targets	FFAH	*In vivo*	(289)
NAGly	Small molecular targets	NA	*In vivo*	(290)
BIIL 284	LTs targets	LTB4R	Phase 1	(291)
Iloprost	PGs targets	PGI2	Phase 2	(292)
GW274150	NO targets	iNOS	Phase 2	(293)

## 4 Cellular and Molecular Targets in Rheumatoid Arthritis

Biological targets may be a protein or nucleic acid involved in a specific biological pathway that may be targeted to design drug molecules.^41^ Molecular targets with crucial functions in RA pathogenesis are mentioned below, with their possible applicability in drug and vaccine development.

### 4.1 Peptidyl-Arginine-Deiminase (PAD)

Peptidyl-arginine-deiminase (PAD) plays a crucial role in RA by facilitating citrullination, which converts protein arginine residues to citrulline. This citrullination process produces autoantibodies against citrullinated proteins called anticitrullinated protein antibodies (ACPA).^42^ ACPA serves as a diagnostic marker for RA and is believed to contribute to disease development and progression by triggering immune responses and promoting joint inflammation. Humans have five tissue-specific PADs, namely PAD1–4 and PAD6, encoded by a single gene. PAD2 is predominantly located in the brain, spleen, secretory glands, and skeletal muscles, while PAD4 is primarily present in granulocytes and white blood cells (WBC). PAD6 is expressed in ovaries, eggs, and early embryos.^43,44^ PAD2 and PAD4 are associated with RA pathogenesis as they are overexpressed in the synovial joints. When proteins are citrullinated beyond normal levels, they may be considered autoantigens due to the expression of neo-epitopes.^45,46^ These are recognized by HLA-DR1 and HLA-DR4 molecules, which ultimately lead to the production of ACPAs.^47^ ACPAs are one of the primary factors present in 70% of RA patients and can be used to diagnose the different pathological phases of RA patients. Immune complexes of citrullinated proteins and ACPAs activate macrophages for further processing and release of TNF-α, which causes synovial inflammation and joint destruction.^48^ Iguratimod (IGU) uses citrullination as a therapeutic target to treat RA and has higher efficacy and safety than the combination of methotrexate and dexamethasone. The expression of PAD2 and PAD4 is suppressed by IGU, resulting in decreased hypercitrullinated proteins. Moreover, IGU also downregulates the neutrophil extracellular trap.^49^ Immunotherapeutic treatments using therapeutic anticitrullinated protein autoantibodies (tACPAs) reduce the immunological burden caused by hyper-citrullinated proteins. These tACPAs target a specific epitope region in the N-terminal sequence of histone-2A. Citrullinated histones are associated with inflammatory neo-epitope expression in the immune system, further producing autoantibodies. Masking these new epitopes by therapeutic antibodies may reduce joint inflammation.^50,51^ Citrullinated proteins may also be a potential target for vaccine design since neo-epitopes of the citrullinated proteins could be targeted before the severe stage of RA by using specific vaccines.^52,53^

### 4.2 Immune Effector Cells

**Figure 5:**
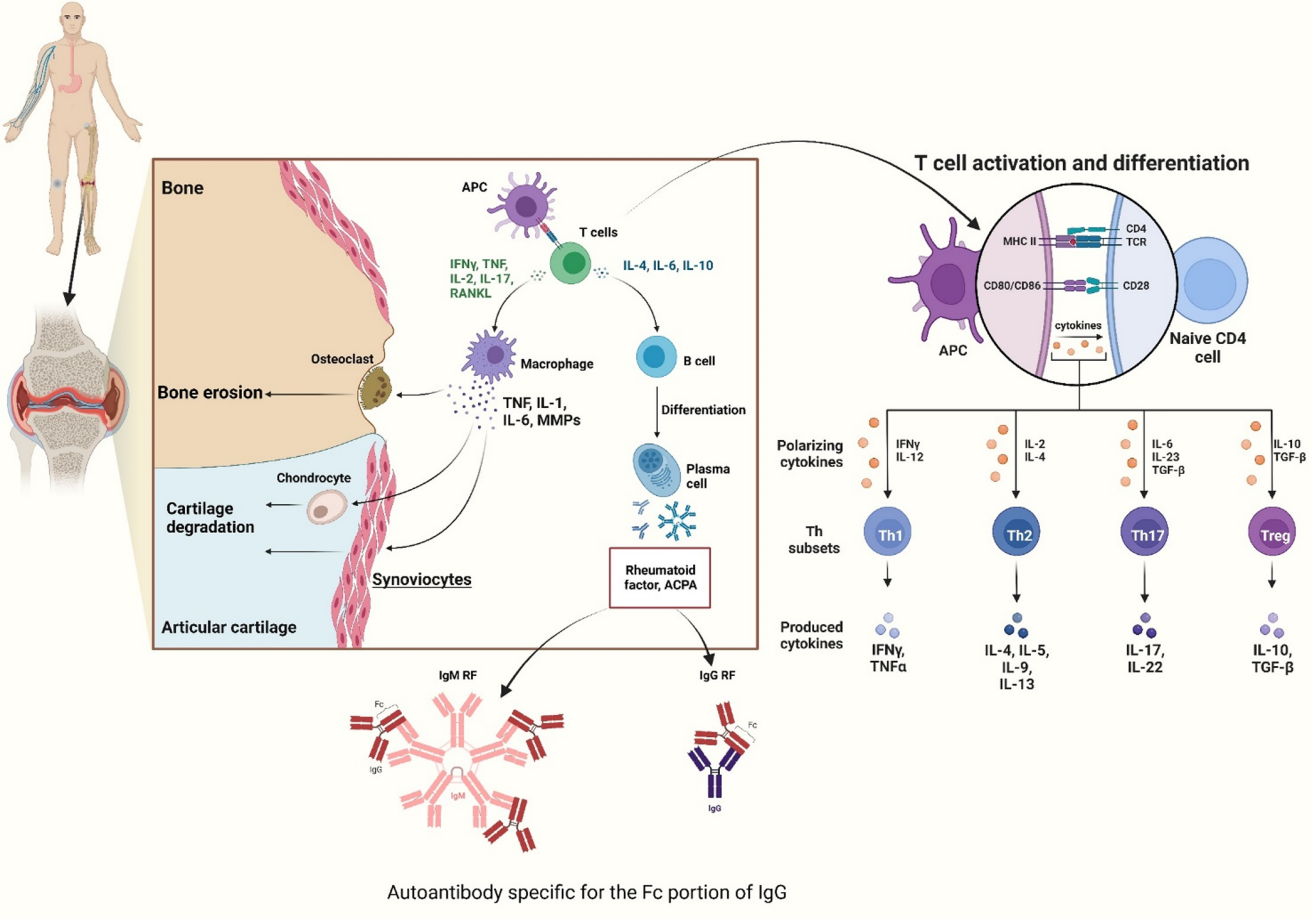
Pathogenesis of RA and the role of T cells, B cells, and IgG antibodies in RF. T cells interact with antigen-presenting cells (APCs) and produce pro-inflammatory cytokines, contributing to RA development. CD4+ T cells, particularly Th1 and Th17 subsets, secrete cytokines (IFN-γ, IL-17, TNF-α) that promote synovial inflammation and joint destruction. Tregs suppress effector T cell activation. B cells produce autoantibodies like RF and anti-CCP antibodies. RF (IgM antibody) recognizes IgG antibodies, forming immune complexes that activate complement and cause synovial inflammation. Anti-CCP antibodies target citrullinated proteins abundant in inflamed synovium and cartilage, contributing to synovial inflammation and joint destruction through immune complex formation (Created using Biorender).

The immune system assumes a dual role in disease processes, acting as a catalyst for recovery while being involved in the progression of autoimmune disorders. RA involves activating innate and adaptive immune systems, mainly immune effector cells (IECs) such as B and T cells, macrophages, neutrophils, NK cells, mast cells, and FLS. In RA, the synovial cavity transitions from an acellular stage to a cellular stage characterized by hyperplasticity and invasiveness. The IECs are primarily located in synovial fluid and related tissues, and their production of pro-inflammatory cytokines contributes to disease signaling through mechanisms that are not yet fully understood (Figure 5**&**Table 3).

**Table 3:** Role of Various Immune Cells and Their Secreted Cytokines in RA Animal Models and Human RA Patients^a^

**Cell type**	**Model**	**Expression of interleukins/cytokines**	**Expression area**	**Functional effect**	**Reference**
Th1	Mouse models	IL-12	Collagen-induced arthritis	Early disease induction and late-stage disease suppression	(294)
Th1 + Treg	Mouse models	TNF-α	TNFα-induced arthritis	Disease progression and Treg dysfunctionality	(295)
Th1	Mouse models	IFN-γ	Pristane-induced arthritis	Early induction of disease	.^296^
Th2	Mouse models	IL-4	Collagen-induced arthritis	Suppression of disease by reducing inflammation and cartilage pathology	(63,297)
Th2	Mouse models	IL-13	Collagen-induced arthritis	Suppression of disease	(298)
Th17	Mouse models	IL-17	Collagen-induced arthritis	Progression and severity of disease	(299,300)
Th17	Mouse models	IL-23	Collagen-induced arthritis	Induction and progression of the disease by promoting Th17 functions	(301)
Tregs	Human	TNFα	Serum	Downmodulates Treg and improves disease severity	(302)
Th1	Human	IL-18	Serum	Upregulated levels in serum	(303)
Th1	Human	IL-15	Serum	Upregulated levels in serum	(303)
Treg	Human	IL-10	RA synovial membrane and serum	Regulation of disease	(304)
Th17	Human	IL-21	Serum	Increases disease severity of RA patients	(305,306)
Th9	Human	IL-9	Serum and synovial tissue	Upregulated levels in serum and synovial tissue	(71,75)

a This table summarizes the important role played by various immune cells and their secreted cytokines in the pathogenesis of RA. Immune cells such as T cells and sub types of T helper cells are involved in the development and progression of the disease.

#### 4.2.1 B Cells

B cells are vital in RA, presenting antigens to activate CD4^+^ T cells and producing pro-inflammatory cytokines (like TNFα).^54^ FLS are mesenchymal cells (type B synovitis) that line the synovial membrane’s inner layers, induce inflammation, and express proteases, which destroy the cartilage and promote an aggressive RA phenotype. Much attention has been focused on B-cell-based therapy due to its involvement in producing Rheumatoid factor and various cytokines. Rituximab (Rituxan), the FDA-approved monoclonal antibody targeting CD20, is effective in targeting and suppressing B cells.^55^ CD20 is absent in stem cells and is lost before B cell differentiation into plasma cells, ensuring the therapy’s specificity.

#### 4.2.2 T Cells

T cells are vital in RA pathogenesis, and the imbalances among different T cell subsets contribute to disease development and progression. Pro-inflammatory factors can disrupt T cell regulation and impact their flexibility. Additionally, T cells are involved in cytokine secretion (IL-1, IL-2, TNFα, and MMPs) associated with RA progression. Different T helper subsets (Th1, Th17, Th9, and Th22) facilitate various stages of the disease from rolling to extravasation into inflamed joints.^56^

##### 4.2.2.1 Th1 and Th2 Cells

Th1 and Th2 cells have distinct roles in the development and progression of RA.^57^ Th1 cells and their cytokines (IFN-γ, TNF-α, IL-1β) are notable in early stages and contribute to the accumulation of dendritic cells and neutrophils in synovium.^58^ In addition, IFN-γ influences disease severity too.^59^ Th1 cells create a pro-inflammatory synovial environment via cytokines, leading to bone/cartilage degradation.^60^ In contrast, Th2 cells have a protective role in the early and late stages of RA. They produce anti-inflammatory IL-4, inhibiting other cytokines like IL-1, TNF-α, IL-8, IL-6, and IL-12 and inhibit bone resorption.^61^ Additionally, IL-4 has been found to delay cartilage damage and significantly reduce the onset of disease and inflammation.^62,63^

##### 4.2.2.2 Th17 Cells

Th17 cells also play critical roles in RA development.^56,64^ Th17 cells are classified into “pathogenic” and “nonpathogenic” types. Pro-inflammatory cytokines (IL-17A, IL-17F, IL-22) are secreted by pathogenic Th17 cells, whereas nonpathogenic Th17 cells have the potential to produce the immunosuppressive cytokine IL-10.^65^ IL-17 triggers the synthesis of pro-inflammatory cytokines (such as TNF-α, IL-1β, and IL-6) in various cells, including cartilage, synovial cells, macrophages, and bone cells. Additionally, it promotes the secretion of chemokines (CXCL1, CXCL2, CXCL8, CCL2, CCL7, CCL20), which recruit neutrophils, macrophages, and lymphocytes to the synovial membrane, contributing to the inflammatory response.^66,67^ Interestingly, the absence of IL-17 has been shown to ameliorate arthritis development.^68^ As a result, IL-17A blockers, such as Secukinumab, Ixekizumab, and Brodalumab are being explored as promising options for treating RA.^69^

##### 4.2.2.3 Th9 Cells

Th9 cells are more abundant in RA patients compared to healthy individuals.^70^ Their upregulation correlated with the accumulation of B and T cells in ectopic lymphoid structures.^71^ In inflammatory arthritis, IL-9 has been found to promote the growth and survival of pathological T cells in the synovium, triggered by mTOR kinase cascade activation. Concurrently, IL-9 levels were observed to be higher in psoriatic arthritis and RA patients compared to osteoarthritis patients.^72^ In RA, there is an elevation of Th9 cells that correlates positively with disease activity by releasing IL-9. Where, IL-9 exerts multiple effects on various cells within the synovium including the enhancement of neutrophil survival and the stimulation of MMP9 production, which contributes to joint inflammation and tissue damage. Moreover, IL-9 facilitates the differentiation of Th17 cells that contribute to autoimmune responses and inflammation. IL-9 also augments the function of synovial Th1 cells modulating immune responses and promoting inflammatory processes.^73,74^ Interestingly, a positive association has been observed between the presence of rheumatoid factor and elevated levels of IL-9 in the serum. Specifically, individuals with two or more isotypes of the rheumatoid factor or positive anti-CCP antibodies are more likely to exhibit higher levels of IL-9.^75^ Vyas et al. observed elevated IL-9 expression in the serum of RA patients compared to healthy individuals, indicating potential involvement of the IL-9/Th9 axis in RA.^76^ Moreover, in RA patients who exhibit poor response to infliximab treatment, the presence of higher levels of Th9 cells contributed to directing the immune response against specific components of the infliximab drug.^77^ Apart from IL-9, IL-23 also regulates the balance of Th17/Th9/Treg cells.^78^

##### 4.2.2.4 Treg Cells

Treg cells maintain immune tolerance, suppress immune activation, and prevent excessive immune responses.^79^ In RA, diminished or dysfunctional Treg cells result in a loss of immune tolerance, leading to an exaggerated immune response against joint tissues.^80^ Specific roles of Treg cells include suppressing autoreactive T cells, regulating pro-inflammatory cytokines, interacting with other immune cells to modulate their function, and inhibiting osteoclastogenesis.^81^ Understanding the role of Treg cells in RA is important for developing targeted therapies to restore their function and maintain immune tolerance.^82^ Therapeutic strategies like Treg cell expansion, adoptive transfer, or modulation of Treg cell activity are being explored to address immune dysregulation and inflammation in RA.^83^ Foxp3 is a key regulator of Treg cell survival.^84^ Its expression is tightly controlled at various levels, including epigenetically, transcriptionally, and post-translationally, and it is vital for maintaining immune self-tolerance.^82,85^ Furthermore, genes like NFAT, STAT5, and Foxo1 interact with the promoter region of Foxp3, regulating its expression.^82,86,87^

### 4.3 JAK-STAT Proteins

The Janus kinase and signal transducer and activator of transcription (JAK-STAT) signaling pathway is involved in RA and contributes to inflammation. Cytokines like IL-6, IL-7, IL-12, and IL-15 bind to type I/II cytokine receptors, initiating downstream signaling and promoting inflammatory events.^88^ Four mammalian JAKs have been extensively studied: JAK1, JAK2, JAK3, and Tyk2 (tyrosine kinase 2), while seven diverse but homologous STAT have been identified: STAT1–4, STAT5a-5b, and STAT6.^89^ STAT protein expression fluctuates based on cell type, activation by different cytokines, and the binding of specific or multiple other ligands. STAT protein function is pleiotropic, depending on cell type, disease stage, and the adjacent inflammatory microenvironment. STAT3 and STAT1 have been implicated in RA pathogenesis.^90^ The activation of STAT3 is predominantly triggered by IL-6 and related cytokines that utilize the gp130 receptor subunit. These cytokines are abundantly expressed in the synovial fluid of individuals with RA.^91^ STAT3 can also activate the STAT1-associated genes STAT4 and STAT6 in RA synovitis. It can also induce inflammation by promoting T-cell survival and antibody production while inhibiting the apoptosis of synovial fibroblasts.^92,93^ Different studies suggest that B-cell antigen receptor and CCR5 may also activate the STAT1 which further stimulates synovial inflammation by inducing the expression of inflammatory genes.^94^ In contrast, there are some contradictory functions of STAT1, such as activation of apoptosis and growth arrest in various cell types, suggesting a protective role in RA. These contradictory functions of STAT proteins mandate further needs research before being considered as therapeutic targets.^95^ Overall, STAT1 may play pathogenic as well as protective roles in RA synovitis. STAT3 may be a novel target in specific cell types, such as synovial fibroblasts and lymphocytes, but not in macrophages, where it is anti-inflammatory.

Tofacitinib, an oral JAK inhibitor (jakinib), can inhibit different isoforms of JAK, primarily JAK1 and JAK3.^96^ Utilization of tofacitinib in RA patients has reduced MMPs and interferon-related genes in the synovium and reduced phosphorylation of STAT1 and STAT3, ultimately down-regulating IL-6 mediated signaling.^97^ Tofacitinib is the first FDA-approved jakinib for treating RA.^98^ Other second-generation inhibitors are also under clinical trials for the treatment of advanced RA. For example, Decernotinib (VX-509) is a next-generation Jakinib undergoing phase II clinical trials and has demonstrated better selectivity for JAK3.^99,100^ Filgotinib is also a next-generation jakinib under phase IIA trials that inhibits the expression of JAK1 and JAK2.^101^ Interstitial lung disease is a pulmonary complication of RA. Resveratrol, a phytoalexin with anti-inflammatory and antioxidant properties targeting the JAK-STAT and RANKL signaling pathways, has been demonstrated to be more specific for RA-related interstitial lung.

### 4.4 Nuclear Factor Kappa B (NF-κB)

NF-κB is a versatile transcription factor in the cytoplasm, activating various genes with diverse functions. NF-κB is important in RA through its involvement in the signaling of cytokines IL-6, IL-1, IL-8, and TNF-α which play a significant role in inflammation.^102^ It also plays a vital role in breaking central and peripheral tolerance, leading to the development of autoimmunity and in the maturation of B, T, and dendritic cells.^103^ The well-known inflammation mediators TNF-α, IL-1, and IL-6, are associated with NF-κB and promote chronic inflammation and joint destruction in RA via autocrine and paracrine signaling.

Five types of NF-κB family members: NF-κB-1 (p50/p105), NF-κB-2 (p52/p100), RelA, RelB, and c-Rel have been reported. After activation, NF-κB forms homodimers or heterodimers and translocates into the nucleus to activate different genes and their downstream signaling. NF-κB is rendered inactive in unstimulated cells by the inhibitor of κB (IκB) and activated by the IκB-kinase (IKK) complexes which degrade IκB via proteasomal degradation and activate NF-κB molecules. The IKK complex comprises different subunits such as IKK1/IKK-α, IKK2/IKK-β, and IKK-γ. Among these three subunits, IKK2 plays the most important role in RA inflammation.^104^ In RA, the activation of specific subunits of NF-κB is observed, with p50 and p65 being extensively studied, particularly in macrophages and synovial fluid.^105^ NF-κB and related regulatory kinases may be potential targets to counteract the hyper-responsiveness in inflammatory cells.

Due to its pivotal role as a signaling mediator in RA, NF-κB represents a profoundly appealing therapeutic target for treating this condition. Different types of therapeutics may directly target NF-κB, and others may indirectly modulate the hyper-responsiveness of different inflammatory cytokines (TNF-α, IL-6, and IL-8). Gaining a comprehensive understanding of the network biology underlying these signaling cascades will provide valuable insights for designing of small molecule inhibitors and monoclonal antibodies as therapeutics. IKKs are among the most promising targets in these pathways.^106^ To date, more than 700 inhibitors of the NF-κB have been developed by various researchers and pharma companies.^107^ FDA-approved drugs that target NF-κBare aspirin (IKK-β inhibitor), docetaxel (suppresses the binding of NF-κB with DNA), simvastatin (inhibits phosphorylation of IκB), bortezomib (blocks the activity of NF-κB), and PBS-I086 (directly inhibits the binding of p65 (RelA)).^108−110^ Overall, NF-κB is an attractive target for designing therapeutics against RA, both in individual and combination modes.

### 4.5 Chemokines

**Figure 6:**
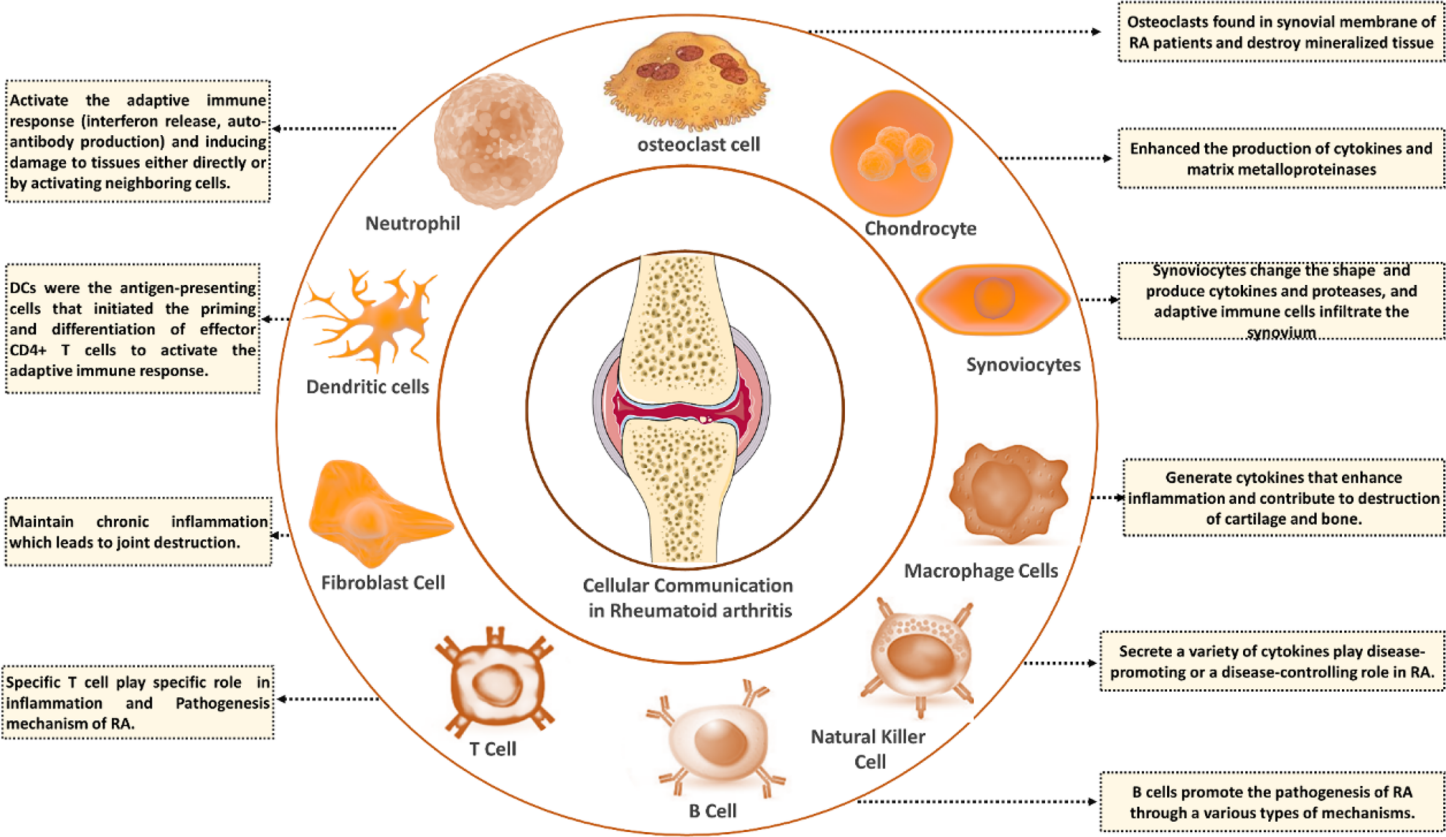
Cellular communication and pathogenic function of immune cells in RA. B cells, T cells, fibroblasts, dendritic cells, neutrophils, osteoclasts, chondrocytes, synoviocytes, NK cells, monocytes, and macrophages contribute to RA pathogenesis. These cells secrete cellular communicators and proteins, including rheumatoid factors (RFs), proinflammatory cytokines, and chemokines, which play a role in the progression and regulation of RA (Created using Biorender).

Chemokines are small proteins that attract immune cells to infection or inflammation sites. They recruit leukocytes to clear infections and regulate immune cell migration and activation. Dysregulation of chemokines can contribute to diseases such as cancer, autoimmune diseases, and inflammatory disorders.^111^ Four subfamilies of chemokines are CXC, C, CC, and CX3C. CXC chemokines mainly recruit neutrophils and have angiogenic properties.^112,113^ They stimulate inflammatory cytokines and MMP3 expression, damaging bone and cartilage. Chemokines also promote synovial hyperplasia.^114,115^ Chemokines act through receptors coupled to GPCRs for signal transduction. CC chemokines attract monocytes, lymphocytes, and immature dendritic cells. CX3C chemokines accumulate neutrophils and lymphocytes.^115^ In RA patients, chemokines are generated by various sources such as synovial tissue, epithelial cells, and subsynovial macrophages^116^ (Figure 6). Cytokines IL-8/CXCL8, ENA-78/CXCL5, GROα/CXCL1, STAP III/CXCL7, and GCP-2/CXCL6 were found elevated in synovial fluid of RA patients. Chemokines P10/CXCL10, Mig/CXCL9, and SDF/CXCL12 were associated with RA under hypoxic conditions.^113^ CC group members MIP3α/CCL20, MCP4/CCL13, MCP1/CCL2, and PARC/CCL18 were also elevated in RA patients. Lymphotactin and RANTES/CCL5 were highly expressed in the pulmonary blood of RA patients.^114^ Fractalkine levels were high in RA synovial fluid.^115^ Synovial cells highly express CXCR2, CXCR3, CCR2, CCR3, and CCR5 in RA patients [^144^].

Targeting chemokines and their receptors holds significant promise in addressing the pathogenesis of RA. For example, Infliximab, an anti-TNFα monoclonal antibody, reduced IL-8/CXCL8 and MCP-1/CCL2 levels.^2^ CP481, a CCR1 antagonist, has shown a 90% decrease in the chemotactic activities in synovial fluid samples of RA patients.^117^ ABX-IL-8, a monoclonal antibody, neutralizes IL-8/CXCL8 and shows promise in treating RA (phase II clinical trials).^72^ Another agent, ABN-912, targets MCP-1/CCL2 chemokine and can potentially be beneficial in RA. Antagonists such as met-RANTES, UCB-35625, and BX-471 target CCR1 and have demonstrated beneficial effects in mitigating disease severity. Additionally, AMD-3100 and 4-F benzoyl TN14003, which target the CXCR4 receptor, are useful in severe RA.^118^ Furthermore, drug molecules like reparixin and SCH-527123 inhibit CXCR1, which is another potential target.^119^ The dysregulation of chemokines is implicated in RA as well as in similar autoimmune diseases and cancer. Targeting chemokines and their receptors using biomolecule therapy shows promise in mitigating disease severity in RA.

### 4.6 Adipokines

Formerly perceived as passive organs primarily responsible for storing energy in the form of triglycerides, adipose tissues have undergone a paradigm shift. They are now acknowledged as endocrine organs that can secrete a wide range of biologically active molecules, collectively called adipokines. These adipokines, encompassing leptin, visfatin, adiponectin, and resistin, assume a key role in regulating insulin resistance and glucose metabolism within our bodies.^120^ The presence of higher adipokine levels in RA, when compared to healthy individuals, indicates a potential relevance of adipokines in the pathogenesis of RA. Adipokines are essential in initiating the release of pro-inflammatory cytokines in affected tissues like synovium, cartilage, and plasma cells, leading to inflammation and promoting a Th1 immune response in RA.^121^ Although numerous studies suggest that adipokines may notably impact RA progression, their exact role in disease development remains uncertain. Leptin, generated by the Lep(ob) gene on chromosome 7q31.3, primarily regulates food intake by stimulating anorexigenic factors and inhibiting orexigenic factors.^122,123^ Through the JAK-STAT signaling pathway, leptin activates synovial fibroblast cells, macrophages, and natural killer cells. This activation process has an impact on the immune response in RA.^124^ Leptin triggers the release of inflammatory cytokines such as IL-1, IL-8, and TNF-α, promoting the production of more leptin. Adiponectin, encoded by a gene on chromosome 3q27, shares similarities with the complement factor C1q.^125^ Adiponectin displays dual characteristics, with the low molecular weight form having anti-inflammatory properties, while the high molecular weight globular form exhibits pro-inflammatory traits.^126^ In RA patients, the upregulated pro-inflammatory form acts through adipoR1 and adipoR2 receptors, producing inflammatory cytokines (IL-8, IL-6, PGE2) and chemokines (MCP1).^127^ Adiponectin also mediates the secretion of matrix metalloproteases (MMP1 and MMP13), destroying the bone matrix and worsening the disease condition.^128^ This synergistic effect contributes to RA progression. In synovial fibroblast tissue, visfatin (PBEF) induces the release of inflammatory cytokines (IL-6, IL-1β, TNF-α) and matrix metalloproteases from synovial fibroblasts and chondrocytes, contributing to joint inflammation and degradation in RA patients.^129^ Resistin, expressed primarily by macrophages, B cells, and plasma cells in the rheumatoid synovium, correlates positively with inflammation markers like CRP, IL-1, and TNF-α. Targeting the vicious cycle of inflammation and adipokine network can lead to substantial beneficial effects of RA.

Adipokine levels can be reduced by blocking IL-6 and TNF-α, which regulate their production. Tocilizumab, an anti-IL-6 monoclonal antibody, reduces pro-inflammatory adipokines and is clinically investigated.^130^ TNF-α blockade molecules (adalimumab, etanercept, and infliximab) lower the levels of adiponectin, visfatin, and resistin, as well as CRP proteins, but do not affect leptin levels.^126^ Inhibiting visfatin with APO866 reduced inflammatory cytokines in the joints and improved disease severity. APO866 has completed phase I trials and is now in phase II clinical investigation.^131^ Contrastingly, DMARDs when used on patients with elevated adipokines, yielded no significant difference in adipokine levels or inflammatory response.^126^ Interestingly, Rituximab, an anti-CD-20 monoclonal antibody that targets visfatin-producing B cells, was also tested but did not change visfatin levels and worsened the disease. Further understanding of adipokine roles is needed to design effective therapeutic strategies in RA.

### 4.7 Adhesion Molecules

Adhesion molecules are proteins that facilitate cell-to-cell interactions and are vital in cell migration, immune response, and embryonic development. The molecular structure of each adhesion molecule is crucial for its function in biological processes.^132^ Cell adhesion molecules include different molecules such as selectins, integrins, ICAM-1 (intercellular cell adhesion molecule), VCAM-1 (vascular cell adhesion molecule), and LFA-1(lymphocyte function-associated antigen). Selectin is a glycoprotein involved in the rolling of the leukocyte by weak interactions before the arrest.^133^ Subsequently, adhesion molecules mediate leukocyte adhesion to endothelial cells for their trafficking from the blood to the tissues.^134^ Adhesion molecules positively correlate with RA progression due to their high expression in synovial endothelial cells, making them a potential therapeutic target for treating RA inflammation.^135^ Compared to the other adhesion molecules, ICAM-1 is more prominent on macrophages, endothelium, and leukocytes in the RA synovium.^136^ Various pro-inflammatory cytokines such as IL-1, IL-6, and TNF-α are also involved in the excess expression of adhesion molecules.^137^ DMARDs such as methotrexate and methylprednisolone decrease adhesion molecule levels in the synovium of RA patients.^138,139^ RA patients administered with infliximab have a reduced concentration of soluble adhesion molecules.^140^ Thalidomide is also an anti-TNF-α agent that can potentially reduce ICAM-1 and LFA-1 levels required for cell–cell interaction.^141^ NSAIDs such as tenidap, PD-I44795, and NCP-15669P2 were reported to be effective in inhibiting the interaction between neutrophils and endothelial cells. They also reduced the expression of adhesion molecules and were found to be well tolerated in clinical evaluations.^142^ SB-27300 is another antagonist against integrin, which is effective in prophylactically reducing the severity of RA destruction.^133^ Vitaxin or MEDI-522, is a humanized monoclonal antibody for neutralizing integrin and is currently under phase II clinical trials.^143^ The anti-ICAM-1 monoclonal antibody Enlimomab, which targets the extracellular domain of this molecule, is effective in treating RA.^144^ Other therapies include the use of carbohydrates which can bind to selectin and inhibit its function in leukocyte rolling, and antisense oligonucleotides which prevent the expression of these adhesion molecules.^145^ Blocking adhesion molecule clustering in RA offers another potential avenue by disrupting the formation of stable adhesion complexes, thereby reducing both leukocyte recruitment and inflammatory cell activation. However, further controlled studies targeting the adhesion molecules are required for assessing the pharmacological modification of hyperinflammatory responses such as that seen in RA.

### 4.8 Enzymes

Enzymes such as MMPs, cathepsins, and serine proteases overexpression leading to excessive degradation of cartilage and bone tissue.^146^ MMPs are responsible for degrading extracellular matrix components such as collagen, gelatin, and proteoglycans.^147^ Cathepsins are lysosomal enzymes that play a role in tissue remodeling and destruction. In RA, they are overexpressed and contribute to the degradation of cartilage and bone tissue.^148^ Serine proteases, such as plasmin and thrombin, are also involved in the pathogenesis of RA. They activate MMPs and other proteases and contribute to the degradation of extracellular matrix components.^149^

#### 4.8.1 Matrix Metalloproteases

MMPs can be categorized into five groups based on substrate specificity: collagenases, stromelysins, adamalysins, matrilysins, and gelatinases.^150^ The other groups of MMP that are not considered in these groups are metalloelastase, enamelysin, and epilysin.^151^ Tissue inhibitors of metalloproteases (TIMPs) regulate MMP activity, and TIMP has shown promise in reducing RA severity. MMP expression is controlled by cis-binding elements like AP-1 and ETS.^152^

Given their active presence in synovial joints, MMPs have emerged as an important target for RA treatment. They contribute to cartilage deterioration and promote angiogenesis in the affected joints.^150,153^ MMPs, including MMP-1, MMP-2, MMP-3, MMP-8, and MMP-9, are produced by synovial lining, neutrophils, macrophages, and chondrocytes.^154^ MMP-1 and MMP-8 are key proteases directly associated with synovial joint inflammation and bone erosion.^154^ Clinical studies are underway to investigate different MMP inhibitors for mitigating RA severity. Batimastat (marimastat), the first developed MMP inhibitor, proved ineffective due to side effects and poor efficacy.^155^ Trocade, a specific collagenase inhibitor, did not clear the phase III clinical trials assessing its efficacy.^156^ Currently, the only MMP inhibitor in use is minocycline, which has also shown limited effectiveness.^157^ GW-3333 and TMI-1 are drug molecules targeting TNF-α Converting Enzyme (TACE), which is overexpressed in RA. However, inhibiting TACE would reduce levels of secreted TNF-α, necessary for an inflammatory response against pathogens.^158^ These inhibitors have demonstrated nonspecificity and side effects like tendinitis, anemia, and musculoskeletal pain, hindering their efficacy.^159^ Future research focuses on identifying more selective inhibitors with minimal adverse effects.

#### 4.8.2 Tyrosine Kinases

The tyrosine kinases (TK) are enzymes capable of transferring phosphate groups to tyrosine (Tyr/Y) residues. The TKs are responsible for maintaining different biological functions such as cellular proliferation, adhesion, cell death, and immune defense. The overexpression of the TKs is associated with angiogenesis and synovial hyperplasia in the RA.^160^ Tyrosine kinase is a highly studied therapeutic target for various inflammatory diseases.^161^ There are multiple inhibitors reported against TKs, such as fostamatinib (spleenTK/STK inhibitor), tofacitinib (a Janus kinase inhibitor), ruxolitinib, evobrutinib, and BMS-986142, but only the first two are being utilized as oral treatments for RA.^162,163^ Further research is needed to understand how TKs modulate the immune response in RA, as their specific mechanism remains unknown.

#### 4.8.3 Mitogen-Activated Protein Kinase (MAPK)

MAPK, belonging to the Ser/Thr kinase family, regulates various cell survival, proliferation, differentiation, apoptotic, and stress responses and mediates the production of inflammatory cytokines.^164^ Three families of MAPK (p38, ERK, and JNK) are prevalent in synovial sites, playing roles in inflammation, tissue remodeling, and destructive enzyme production.^165^ Inhibition of p38 has been shown to reduce bone loss and cartilage destruction. However, clinical trials of p38 inhibitors like VX-702 had limitations in controlling chronic inflammation and producing side effects.^166^ The JNK family stimulates the production of destructive enzymes such as MMPs via MIF and IL-1 and is also involved in inflammatory processes.^167^ MAPK is one of the key stimulators of pro-inflammatory cytokines, mediating inflammation and disrupting the joints in FLS. In macrophages stimulated by LPS (lipopolysaccharide), MAPKs have a profound effect on the levels of TNFα secretion. In the past few years, this function of MAPK has gained attention as a potential molecular target in RA. Inhibition of p38 by SC409 and Org48762–0 was associated with a reduction in bone loss and the number of osteoclasts The p38 inhibition of SD-282 also reduced the serum cartilage oligomeric protein which is a key indicator of cartilage destruction.^168^ VX-702 is a new p38 inhibitor that had promising phase I findings but underperformed in phase II due to its inability to control chronic inflammation, and there were several side effects when compared to patients receiving a placebo.^169^ CI-1040 and SC-236 selectively inhibit ERK phosphorylation, leading to decreased disease symptoms. An inhibitor of JNK(SP600125) has reduced joint destruction in mouse models.^166^ However, none of the inhibitors have shown the expected efficacy and safety required for commercial use. The reason for the inadequate clinical trials is the essential role that these MAPKs have in important cellular processes. Another postulated reason is that most of these inhibitors compete for the ATP binding site, which means they can also nonspecifically inhibit other critical kinase molecules.^164^ Another promising approach is to use a peptide to interfere with the JNK signaling pathway, but this has not yet been tested in an animal model.

Mitogen-activated protein kinase phosphatase (MKP) emerges as a potential therapeutic target for blocking MAPK activation in RA. There are ten forms of MKP in mammals (MKP1–7, DUSP2, HVH3, and HVH5) with different subcellular locations and biochemical characteristics.^170^ MKP, specifically MKP5 or DUSP10, inhibits the activation of AP-1, which regulates the expression of pro-inflammatory genes. Decreased MKP expression in synovial fibroblast-like cells (FLS) correlates with increased disease severity, highlighting the role of MKP in inhibiting pro-inflammatory cytokine expression by blocking MAPK activation.^171^ Glucocorticoids (GC), known for their anti-inflammatory properties, increase MKP gene expression by dimerizing with GC receptors in the cytosol, thereby inactivating MAPK and attenuating the inflammatory response. However, the use of GC is complicated due to irreversible side effects such as hypertension, obesity, and immune compromise.^172^

#### 4.8.4 Histone Deacetylases

The process of acetylation and deacetylation of histone protein is a prominent transcriptomic regulation that allows the activation or deactivation of several genes and is regulated by two enzymes, histone acetylase (HAC) and histone deacetylase (HDAC) enzymes. In RA, HDAC is known to regulate the activation of proinflammatory genes. It is reported that in RA patients, the balance of HAC and HDAC is shifted toward the acetylation of histone proteins. Hence, these hyperacetylated histones cannot stop the activation of proinflammatory genes, ultimately promoting inflammation in RA patients.^173^ There are different types of HDAC: class I includes HDAC-1, 2, 3, and 8, and class II includes HDAC-4, 5, 6, 7, 9, and 10. Modulating the inflammatory process in RA may be possible by using a particular HDAC as a therapeutic target.

Regarding the specificity of HDACs, HDAC-3, and HDAC-6 are highly specific as therapeutic targets. The importance of targeting HDAC-3 relates to its role in regulating nonhistone proteins such as NF-κB, RelA, and cell cycle inhibitors (p21 and p53).^174^ Two HDAC-3 inhibitors, trichostatin A (TSA) and MI192, have shown their effective inhibitory response for HDAC-3. HDAC-6 is highly overexpressed in RA patients, so it can also be a potential target to overcome the inflammatory response. CKD-506 is a selective inhibitor of HDAC-6 and induces the acetylation of tubulin to reduce the effect of HDAC-6.^175^ With a precise selection of the type of HDAC, they may be utilized as a therapeutic option for treating RA. For the inhibition of HDACs, effective inhibitors or blockers or any monoclonal antibody can be designed. Multitarget inhibitors can be synthesized with a more structural understanding, which can hit more than one HDAC (Table 4).

**Table 4:** Drugs, Prodrugs, and Their Epigenetic Targets in RA

**Agent**	**Epigenetic process**	**Target**	**Phases**	**References**
Azacitidine	DNA methylation	DNA methyltransferase	*In vivo*	(307)
Decitabine	DNA methylation	DNA methyltransferase	*In vivo*	(308)
Procainamide	DNA methylation	DNA methyltransferase	*In vivo*	(309)
Hydralazine	DNA methylation	DNA methyltransferase	*In vivo*	(310)
EGCG	DNA methylation	DNA methyltransferase	*In vivo*	(311)
Delphinidin	Histone modification	Histone acetyltransferase	*In vitro*	(312)
EZH2	Histone modification	Histone methyltransferase	*In vitro*	(313)
Entinostat	Histone modification	Histone deacetylases	*In vitro*	(314)
MI192	Histone modification	Histone deacetylases	*In vitro*	(314)
Trichostatin A	Histone modification	Histone deacetylases	*In vivo*	(314)
Vorinostat	Histone modification	Histone deacetylases	*In vitro*	(314)
Nicotinamide	Histone modification	Histone deacetylases	*In vitro*	(315)
Largazole	Histone modification	Histone deacetylases	*In vitro*	(316)
Givinostat	Histone modification	Histone deacetylases	*In vitro*	(317)
Anacardic acid	Histone modification	Histone acetyltransferase	*In vivo*	(318)
GSK-J4	Histone modification	Histone methyltransferase	*In vivo*	(319)
MS-275	Histone modification	Histone deacetylases	*In vivo*	(320)
Valproic acid	Histone modification	Histone deacetylases	*In vivo*	(314)
MPT0G009	Histone modification	Histone deacetylases	*In vivo*	(314)
CKD-506	Histone modification	Histone deacetylases	*In vivo*	(321)
CKD-L	Histone modification	Histone deacetylases	*In vivo*	(322)
NK-HDAC-1	Histone modification	Histone deacetylases	*In vivo*	(323)
SAHA	Histone modification	Histone deacetylases	*In vivo*	(320)
I-BET151	Histone modification	Bromodomain and extra-terminal	*In vivo*	(324)
JQ1	Histone modification	Bromodomain and extra-terminal	*In vivo*	(325)
–	RNA methylation	Methyltransferase-like 3	*In vitro*	(326)

### 4.9 Receptors

#### 4.9.1 Toll-like Receptors (TLRs)

TLRs may connect the complex signaling of RA with a new understanding of disease pathogenesis. Although TLRs are associated with the recognition of microbial pathogens, various studies suggest that endogenous ligands are also processed by the TLRs, such as antibody-DNA complexes, heat shock proteins (HSPs), hyaluronan oligosaccharides, and fibronectin fragments.^176^ The reason TLRs are involved in RA is their structural similarities to the IL-1R family. Signaling induced by different TLRs activates MyD88-dependent signaling pathways and secretion of pro-inflammatory cytokines (IL-6, IL-1). TLR-2, TLR-3, TLR-4, TLR-8, and TLR-9 are mainly linked with RA pathogenesis.^177^ TLR-2 and MyD88 knockout mice were protected from the development of joint swelling in streptococcal cell wall-induced RA.^178^ Arthritis was also reduced in TLR-4 and TLR-9 knockout mice.^179^ TLR-2 and TLR-9 are expressed in synovial fibroblasts and induce the secretion of IL-6 and IL-8.^179^ At the same time, TLR-4 is associated with IL-17 secretion.^180^ TLRs are recognized as second hits in the inflammatory progression of the RA, with the first hit being citrullinated proteins, HLA-DR4, and rheumatoid factors/RF. Their defective function with various ligands supports the severity of RA.^181^

The instrumental role of TLRs in the pathology of RA suggests they may be good targets for treating RA. Strategies to utilize TLRs as potential therapeutic targets involve the suppression of endogenous TLR ligands, blocking downstream pathways, and preventing TLR dimerization by utilizing monoclonal antibodies or inhibitors. Among these, NI0101 is a monoclonal antibody against TLR-4, that is in the preclinical phase, which prevents dimerization and activation of TLR-4. It has also been linked to decreased joint inflammation.^182^ A monoclonal antibody against TLR-2, OPN301, can reduce proinflammatory cytokines in the synovium tissue.^183^ Interestingly, Hydroxychloroquine, used to treat RA, downregulates TLR-9-mediated differentiation of B cells.^180^ In conclusion, a deep understanding of the TLRs found in the synovium of RA patients may open new doors for novel RA therapeutic approaches.

#### 4.9.2 Receptor for Advanced Glycation End Products (RAGE)

RAGE is a member of the superfamily of cell surface receptors with an immunoglobulin-type structure. It comprises one V (variable) type immunoglobulin domain and two C (constant) type domains. It is a multiligand receptor for advanced glycation end products (AGE), which are irreversible products derived from nonenzymatic glycation of reducing sugars and proteins, e.g. carboxymethyl AGE and prenyl glycine AGE.^184^ Other RAGE ligands are HMGB-1 (high mobility group box chromosomal protein-1), also called amphoterin, S-100/calgranulin, and mac-1.^185^ In regular physiology, these ligands play a crucial role in the outgrowth and differentiation of neuritis and stress conditions.^186^ The association of these ligands with the RAGE triggers the activation of diverse signaling cascades involving the MAPK and NF-κB pathways and secretion of inflammatory soluble mediators.^164^ The downstream activation of STAT3, SAPK/JNK, and ERK1/2 signaling depends on cell type and environmental conditions.^187^ These signaling events mediate the secretion of IL-6, IL-1β, TNF α, and MMPs, further increasing oxidative stress and amplifying inflammation.^188^

Advanced oxidation products also bind to this receptor and activate signal transduction pathways. The levels of this receptor are generally low and are only elevated at the site of injury. RAGE expression in lymphocytes and dendritic cells also suggests its possible role in the adaptive immune response.^189^ The receptor has been associated with RA pathogenesis due to its increased expression in RA-FLS and synovial macrophages, leading to synovial hyperplasia. Not only the receptor but the levels of ligands are also upregulated in the synovial joints of RA patients. RAGE overexpression is stimulated by cytokine factors such as IL-1β and IL-17 The severity of disease progression depends on a different form of RAGE, as in the case of 82S polymorphic form that interacts more strongly with its ligand and enhances the pro-inflammatory response in RA patients.^190^ RAGE and its ligand can be possible molecular targets in RA therapies. The receptor can be blocked by using a soluble decoy receptor. The decoy receptor results from alternate splicing of endogenous secretory RAGE (esRAGE) mRNA and can also be generated from the action of a disintegrin and metallopeptidase (ADAM10).^191,192^ This soluble form attenuates the inflammatory mediators preventing tissue damage and inhibiting MMP activation.^186,193^ siRNA-targeted therapy has also been tested against RAGE, resulting in a reduction of inflammation.^187^ The binding of RAGE and its ligands has also been inhibited using a peptide called RAP (RAGE antagonist peptide), which reduces NF-κB activation and its inflammatory response.^187^ Antibodies against RAGE ligands, such as S100A12 and HMGB-1, are also potential treatment strategies for RA.^188,194^ TNF-α also stimulates S100A12 expression; the anti-TNFα antibody infliximab decreased the levels of the ligand S100A12 in both *in vivo* and *in vitro* experiments.^188^ Targeting these signaling events might result in future therapeutic strategies for treating RAGE-mediated RA pathogenesis.

## 5 Noncoding RNAs and Their Role in Rheumatoid Arthritis

### 5.1 miRNAs

**Figure 7:**
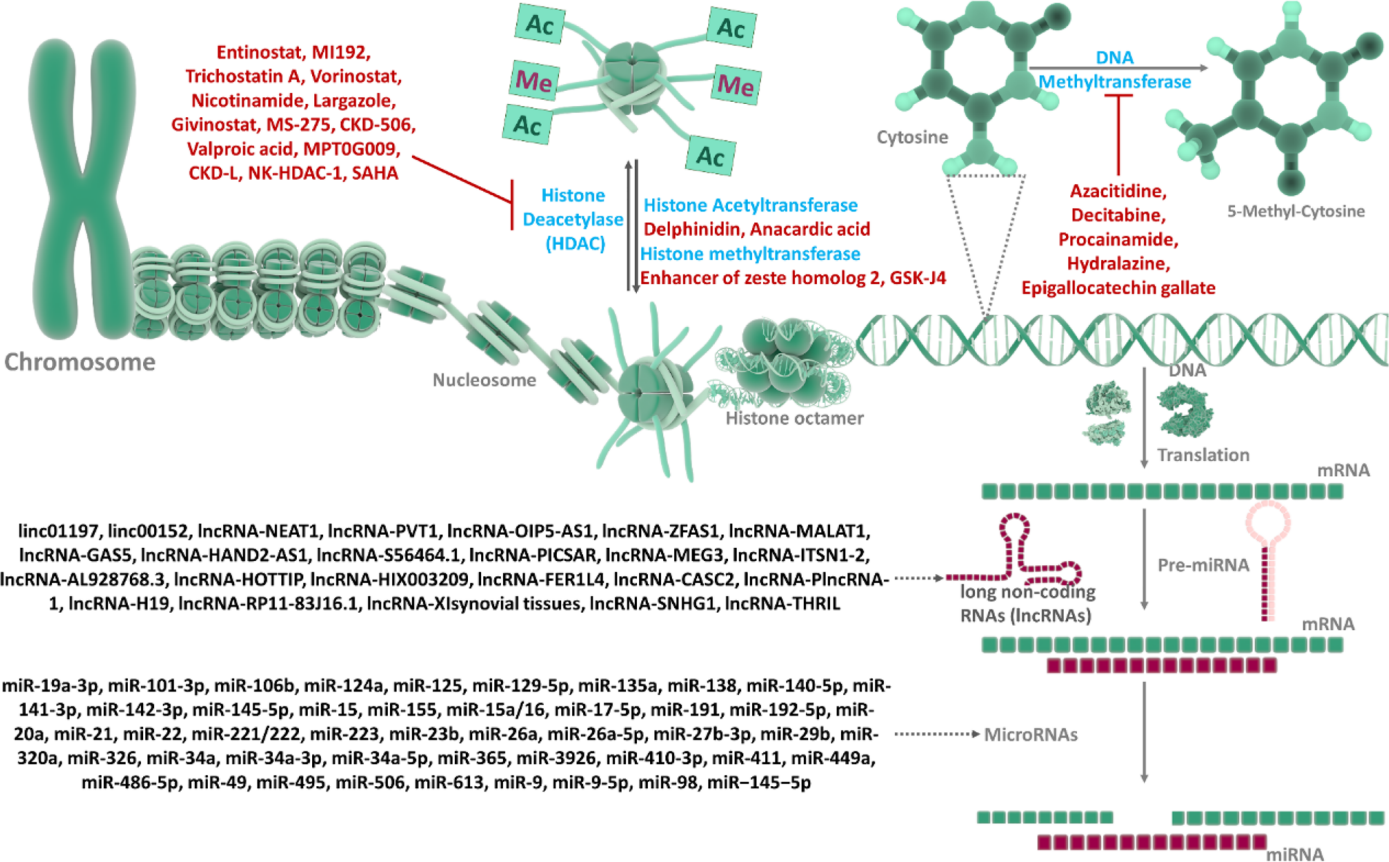
Regulatory role of prodrugs, drugs, epigenetic targets, noncoding RNAs, and their targets in RA pathogenesis. Prodrugs, drugs, and epigenetic targets can modulate gene expression related to inflammation, autoimmunity, and bone remodeling. They target specific proteins, enzymes, and pathways implicated in RA pathogenesis, including cytokines, chemokines, transcription factors, and histone deacetylases. Noncoding RNAs (microRNAs and long noncoding RNAs) regulate gene expression and are involved in RA pathogenesis. They target specific mRNAs, influencing their stability and translation, and thereby regulating protein expression (Created using Biorender).

MicroRNAs (miRNAs) are short noncoding endogenous RNAs, mostly found in biological fluids such as plasma and urine. These miRNAs regulate the level of different mRNAs in various cellular processes. Numerous experimental studies indicate an association between the dysregulated miRNA expression and RA pathophysiology.^195−198^ Various miRNAs have been reported as prediagnostic biomarkers and therapeutic targets in RA.^199^ The miRNA-146a and miRNA-155 are found to be associated with RA. The expression miRNA-146a is upregulated in the synovial fluid of RA.^200^ In RA patients, miRNA-155 has been observed to be upregulated in macrophages present in the synovial fluid. This miRNA is involved in T and B cell differentiation processes. More details about various RA-related miRNAs have been mentioned elsewhere^201^ (Figure 7**&**Table 5).

**Table 5:** Noncoding RNAs and Their Target Signaling Pathways in RA

**NcRNAs**	**Expression**	**Tissue**	**Signaling**	**Phases**	**References**
**Micro RNAs (miRNAs)**					
miR-138	Upregulation	Fibroblast-like synoviocytes	NF-kB signaling	*In vitro*	(327)
miR-34a-3p	Downregulation	Fibroblast-like synoviocytes	–	Animal synovial tissue study	(328)
miR-23b	Upregulation	Fibroblast-like synoviocytes, synovial tissues	–	–	(258)
miR-125	Downregulation	Synovial tissues	PI3K/Akt/mTOR pathway	*In vitro*	(329)
miR-27b-3p	Downregulation	Synovial tissues	HIPK2 signaling	*In vitro*	(330)
MiR-19a-3p	Upregulation	Synovial tissues	IGFBP5 signaling	*In vitro*	(331)
MiR-19a-3p	Downregulation	Plasma	SOCS3	*In vitro*	(332)
miR-142–3p	Upregulation	Synovial tissues, Fibroblast-like synoviocytes	NF-kB signaling	*In vitro*	(333)
miRNA-135a	Upregulation	Synovial tissues	PI3K/AKT pathway	*In vitro*	(334)
miR-192–5p	Downregulation	BMMSC-exos	–	–	(335)
miR-98	Upregulation	Fibroblast-like synoviocytes	IL-10 signaling	*In vitro*	(336)
miR-129–5p	Downregulation	Fibroblast-like synoviocytes	IGF-1R/SRC/ERK/EGR-1 pathway	*In vitro*	(337)
miR-26a-5p	Upregulation	Fibroblast-like synoviocytes	PTEN/PI3K/AKT pathway	*In vitro*	(338)
miR-221/222	Upregulation	Peripheral blood mononuclear cell	–	–	(339)
miR-191	Upregulation	Fibroblast-like synoviocytes	miR-191-C/EB peripheral blood pathway	*In vitro*	(340)
miR-449a	Downregulation	Synovial tissues	HMGB1 signaling	*In vitro*	(341)
miR-410–3p	Downregulation	Synovial tissues, Fibroblast-like synoviocytes	NF-kB signaling	*In vitro*	(342)
miR-506	Downregulation	Synovial tissues, Fibroblast-like synoviocytes	TLR4 signaling	*In vitro*	(343)
miR-320a	Downregulation	Synovial tissues	MAPK-ERK1/2 pathway	*In vitro*	(344)
miR-29b	Upregulation	Peripheral blood	HBP1 signaling	*In vitro*	(345)
miR-155	Upregulation	Synovial tissues	FOXO3a signaling	*In vitro*	(346)
miR–145–5p	Upregulation	Fibroblast-like synoviocytes	NF-kB pathway	Animal synovial tissue study	(347)
miR-22	Downregulation	Fibroblast-like synoviocytes	IL6R signaling/NF-kB pathway	*In vitro*	(348)
miR-22	Downregulation	synovial tissues	SIRT1 signaling	*In vitro*	(349)
miRNA-141–3p	Downregulation	Fibroblast-like synoviocytes	FoxC1/b-catenin axis	Animal culture	(350)
miR-101–3p	Downregulation	Fibroblast-like synoviocytes	PTGS2 signaling	*In vitro*	(351)
miR-495	Downregulation	Fibroblast-like synoviocytes	b-catenin pathway	*In vitro*	(352)
miRNA-17–5p	Downregulation	Fibroblast-like synoviocytes	JAK/synovial tissues AT pathway	Animal synovial tissue study	(353)
miRNA-140–5p	Downregulation	Fibroblast-like synoviocytes	synovial tissuesAT3 signaling	*In vitro*	(354)
miR-3926	Downregulation	Fibroblast-like synoviocytes	TLR5 signaling	*In vitro*	(355)
miR-613	Downregulation	Fibroblast-like synoviocytes, synovial tissues	DKK1 signaling	*In vitro*	(356)
miR-15	Upregulation	Fibroblast-like synoviocytes	NF-kB pathway	*In vitro*	(357)
miR-21	Downregulation	Fibroblast-like synoviocytes	Wnt pathway	Animal synovial tissue study	(358)
miRNA-15*a*/16	Downregulation	Fibroblast-like synoviocytes	SOX5 axis	*In vitro*	(359)
miRNA-155	Upregulation	Fibroblast-like synoviocytes	–	–	(360)
miR-26a	Downregulation	Cartilage tissues, articular chondrocytes	Cartilage tissues GF signaling	Animal synovial tissue study	(361)
miR-106b	Downregulation	Synovial fibroblast-derived exosomes	PDK4 signaling	*In vitro*	(362)
miR-223	Upregulation	Fibroblast-like synoviocytes	–	–	(363)
miR-411	Downregulation	Synovial tissues, Fibroblast-like synoviocytes	NF-kB pathway	Animal synovial tissue study	(364)
miR-9	Downregulation	Fibroblast-like synoviocytes	NF-kB1-RANKL pathway	Animal synovial tissue study	(365)
miRNA-486–5p	Downregulation	Fibroblast-like synoviocytes-exos	Tob1/BMP/Smad pathway	Animal synovial tissue study	(366)
miR-49	Upregulation	Peripheral blood mononuclear cell	–	–	(367)
miR-326	Downregulation	Peripheral blood mononuclear cell	–	–	(367)
miR-34a-5p	Downregulation	Synovial tissues	XBP1 signaling	*In vitro*	(368)
miR-20a	Downregulation	Fibroblast-like synoviocytes	ADAM10 signaling	*In vitro*	(369)
miR-145–5p	Downregulation	Fibroblast-like synoviocytes	Wnt1/b-catenin pathway	*In vitro*	(370)
miR-365	Downregulation	Fibroblast-like synoviocytes	IGF1 signaling or PI3K/AKT/mTOR pathway	Animal synovial tissue study	(371)
miR-34a	Downregulation	BM-MSC-Evs	cyclin I/ATM/ATR/p53 axis	*In vitro*	(372)
miR-124a	Downregulation	Fibroblast-like synoviocytes	PIK3/NF-kB pathway	*In vitro*	(373)
miR-9–5p	Downregulation	Serum	RE synovial tissues/miR-132 pathway	*In vitro*	(374)
miR-34a-5p	Downregulation	Synovial tissues	XBP1 signaling	*In vitro*	(368)
**Long noncoding RNAs (lncRNAs)**					
linc01197	Downregulation	Synovial tissues	miRNA-150/THBS2 axis	*In vitro*	(375)
lncRNA NEAT1	Upregulation	Peripheral blood mononuclear cell-exos	miRNA-23a/MDM2/SIRT6 Axis	*In vitro*	(376)
lncRNA NEAT1	Upregulation	Synovial tissues, Fibroblast-like synoviocytes	miR-204–5p signaling	*In vitro*	(377)
lncRNA NEAT1	Upregulation	Synovial tissues, Fibroblast-like synoviocytes	MAPK/ERK pathway	*In vitro*	(378)
lncRNA NEAT1	Upregulation	Fibroblast-like synoviocytes	miR-410–3p/YY1 axis	*In vitro*	(379)
lncRNA PVT1	Upregulation	Fibroblast-like synoviocytes	miRNA-145–5p	*In vitro*	(380)
lncRNA PVT1	Upregulation	Synovial tissues	miR-543-dependent SCUBE2	*In vitro*	(381)
lncRNA PVT1	Upregulation	Fibroblast-like synoviocytes	SIRT6	*In vitro*	(382)
lncRNA OIP5-AS1	Downregulation	Fibroblast-like synoviocytes	miR-448-PON1/TLR3/NF-kB axis	*In vitro*	(383)
lncRNA ZFAS1	Upregulation	Fibroblast-like synoviocytes	miR-296–5p/MMP-15	Animal synovial tissue study	(384)
lncRNA ZFAS1	Upregulation	Fibroblast-like synoviocytes	miR-2682–5p/ADAMTS9 axis	*In vitro*	(385)
linc00152	Upregulation	Fibroblast-like synoviocytes	Wnt/b-catenin pathway	*In vitro*	(386)
lncRNA MALAT1	Downregulation	Peripheral blood mononuclear cell	Notch pathway	*In vitro*	(387)
lncRNA GAS5	Downregulation	Fibroblast-like synoviocytes	miR-128–3p/HD articular chondrocytes4 axis	*In vitro*	(388)
lncRNA GAS5	Downregulation	Synovial tissues, fibroblast-like synoviocytes	HIPK2 signaling	*In vitro*	(389)
lncRNA HAND2-AS1	Downregulation	Mesenchymal stem cell-derived exosomes	miR-143–3p/TNFAIP3/NF-kB pathway	*In vitro*	(390)
lncRNAS56464.1	Upregulation	Fibroblast-like synoviocytes	miR–152–3p/Wnt pathway	*In vitro*	(391)
lncRNA PICSAR	Upregulation	Fibroblast-like synoviocytes	miRNA-4701–5p signaling	*In vitro*	(392)
lncRNA MEG3	Downregulation	Fibroblast-like synoviocytes	miR-141/AKT/mTOR pathway	Animal synovial tissue study	(393)
lncRNA ITSN1–2	Upregulation	Fibroblast-like synoviocytes	NOD2/RIP2 pathway	*In vitro*	(394)
lncAL928768.3	Upregulation	Fibroblast-like synoviocytes	–	–	(395)
lnc-AC091493.1	Upregulation	Fibroblast-like synoviocytes	–	–	(395)
lncRNA HOTTIP	Upregulation	Fibroblast-like synoviocytes	synovial tissuesRP1 demethylation	*In vitro*	(396)
lncRNA HIX003209	Upregulation	peripheral blood mononuclear cell	TLR4/NF-kB pathway	*In vitro*	(397)
lncRNA FER1L4	Downregulation	synovial tissues, Fibroblast-like synoviocytes	NLRC5 signaling	*In vitro*	(398)
lncRNA CASC2	Downregulation	Plasma	IL–17 signaling	*In vitro*	(399)
lncRNA PlncRNA-1	Downregulation	Serum, synovial tissues	TGF-b1 signaling	*In vitro*	(400)
lncRNA H19	Upregulation	Fibroblast-like synoviocytes	Notch pathway	*In vitro*	(401)
lncRNA H19	Upregulation	Fibroblast-like synoviocytes	miR-124a	Animal synovial tissue study	(402)
lncRNA RP11–83J16.1	Downregulation	Fibroblast-like synoviocytes	b-catenin pathway	*In vitro*	(403)
lncRNA H19	Downregulation	Fibroblast-like synoviocytes	NF-kB and JNK/p38 MAPK pathways	*In vitro*	(404)
lncRNA XI	Upregulation	Cartilage tissues	synovial tissuesAT3 signaling	Animal synovial tissue study	(405)
lncRNA SNHG1	Upregulation	Fibroblast-like synoviocytes	PTBP1 signaling	*In vitro*	(406)
lncRNA THRIL	Upregulation	Serum	PI3K/AKT pathway	*In vitro*	(407)
**Circular RNAs (circRNAs)**					
circ_0088036	Upregulation	Fibroblast-like synoviocytes	miR-140–3p/SIRT 1 axis	*In vitro*	(408)
circ_0000396	Downregulation	Fibroblast-like synoviocytes	miR-203/HBP1 axis	*In vitro*	(217)
circ_AFF2	Upregulation	Fibroblast-like synoviocytes	miR-375/TAB2 axis	*In vitro*	(409)
circ_0130438	Downregulation	Peripheral blood mononuclear cell	–	–	(410)
circ_0002715	Upregulation	Peripheral blood	–	–	(411)
circ_0035197	Upregulation	Peripheral blood	–	–	(411)
circRNA_09505	Upregulation	Peripheral blood mononuclear cell	miR-6089/AKT1/NF-kB axis	Animal synovial tissue study	(412)
circFADS2	Downregulation	Articular chondrocytes	miR-498/mTOR pathway	*In vitro*	(413)
circ_0000175	Downregulation	Peripheral blood mononuclear cell	–	–	(414)
Circ_0008410	Upregulation	Peripheral blood mononuclear cell	–	–	(414)

### 5.2 Long Noncoding RNAs

Long noncoding RNA (lncRNA) is a class of RNA molecules that are longer and do not code for proteins. They play diverse roles in cellular processes, gene regulation, and disease development.^202,203^ Multiple studies have indicated the involvement of lncRNAs in the development and progression of RA.^204−206^ For instance, one study identified the upregulation of lncRNA H19 in the synovial tissue of RA patients.^207^ Another study demonstrated the downregulation of lncRNA GAS5 in PBMC of RA patients which was associated with the inhibition of cell proliferation and activation.^208,209^ Furthermore, lncRNAs have also been identified as regulators of the expression of various cytokines and chemokines that hold crucial roles in the pathogenesis of RA.^210^ The lncRNA NEAT1 was found to promote the expression of pro-inflammatory cytokines.^211^ Overall, these findings suggest lncRNAs have significant involvement in the development and progression of RA, and targeting specific lncRNAs could potentially offer a novel therapeutic approach for the disease.

### 5.3 Circular RNAs

Circular RNAs (circRNAs) are noncoding RNA molecules formed by back-splicing of exons or introns. They regulate gene expression by interacting with miRNAs as sponges or RNA-binding proteins.^212,213^ Numerous studies have explored the involvement of circRNAs in the development and progression of RA.^214,215^ Ouyang et al. found significantly increased expression levels of circRNA_104871, circRNA_003524, circRNA_101873, and circRNA_103047 in PBMCs of RA patients compared to healthy individuals.^216^ These circRNAs were found to enhance the proliferation and migration of fibroblast-like synoviocytes.^214^ A decreased expression of circRNA_0000396 and circRNA_0130438 in PBMCs of RA patients compared to healthy controls was observed. Additionally, these circRNA were found to regulate the expression of multiple genes involved in immune system regulation and inflammation.^217^

## 6 Gut Microbiota in Rheumatoid Arthritis

The gut microbiota plays a crucial role in immune dysfunction, with alterations in its composition and function contributing to the disease progression. Reyes-Castillo^218^ and Zhao^219^ both highlight the potential of the gut microbiota as a diagnostic and therapeutic target for RA, where Reyes-Castillo specifically discusses the molecular mechanisms involved. Tsetseri^220^ and Li^221^ further emphasize the role of gut dysbiosis in RA, where Tsetseri proposes three potential mechanisms and Li focuses on the influence of the gut microbiota on gut homeostasis and RA. In high-risk individuals for RA, gut microbiota dysbiosis has been shown to trigger mucosal immunity perturbation and promote arthritis in mice, further supporting the role of gut microbiota in RA pathogenesis.^222^ Research has shown a strong link between gut microbiota and RA, with alterations in gut microbiota composition and function contributing to the development and progression of the disease.^218,223^ Specific bacteria, such as Prevotella species, have been found to be dominant in the intestines of preclinical RA patients, and their presence has been linked to the development of Th17 cell-dependent arthritis.^224^ Metagenomic studies have further revealed significant differences in the gut and oral microbiome of RA patients, which can potentially be used for disease stratification and diagnosis.^225^

## 7 Challenges in Rheumatoid Arthritis Treatment

Despite significant advancements in the treatment of RA, current treatment strategies still face several limitations and drawbacks. These challenges encompass drug resistance, adverse effects, and the necessity for personalized medicine approaches.

### 7.1 Drug Resistance

The development of drug resistance poses a significant challenge in treating RA. Certain patients may not respond satisfactorily when treated with conventional DMARDs, such as methotrexate or sulfasalazine. Additionally, patients may resist biologic DMARDs, such as TNF or IL-6 inhibitors.^226,227^ This necessitates the exploration of alternative treatment options, combination therapies, or higher doses, which may not always be effective for all patients.

### 7.2 Adverse Effects

RA treatment often involves using immunosuppressive medications, which can have significant adverse effects. Biologic DMARDs carry an elevated risk of infections and malignancies.^228^ Prolonged usage of corticosteroids can contribute to the development of osteoporosis, weight gain, hypertension, and other metabolic side effects.^229^ NSAIDs can cause gastrointestinal bleeding, kidney damage, and cardiovascular complications.^230^ Common adverse effects of NSAIDs, corticosteroids, DMARDs, and biologic agents medications may include gastrointestinal issues, kidney and liver damage, increased risk of infections, cardiovascular events, weight gain, osteoporosis, mood changes, and neurological issues.^231^ These adverse effects may limit the long-term use of these medications and impact patient well-being overall.

### 7.3 Need for Personalized Medicine Approaches

RA is a heterogeneous disease, and patients exhibit significant variability in disease severity, response to treatment, and underlying molecular mechanisms. Current treatment strategies often follow a trial-and-error approach, where patients are prescribed standard therapies without considering their characteristics. Personalized medicine approaches, such as pharmacogenomics and biomarker-guided treatment, can help to identify specific medications or those at higher risk of adverse effects.^232,233^ Implementing these approaches could improve treatment outcomes and minimize unnecessary exposure to ineffective or harmful medications.

### 7.4 Access and Affordability

The high cost of many RA medications can limit access to optimal treatment for some patients. Biologic DMARDs, in particular, are often expensive and may not be affordable for everyone, leading to disparities in treatment access and outcomes.^234,235^ Addressing the cost barriers associated with these medications is crucial to ensuring all patients can access effective treatment options.

## 8 Future Perspectives of Therapeutic Development and Immunological Studies in Rheumatoid Arthritis

There are several potential future directions for RA therapeutic development and immunological studies. First and foremost, gaining a deeper understanding of the immunological mechanisms involved and the diverse subtypes of RA is essential to developing personalized treatment approaches. Identification of biomarkers for patient stratification and predicting treatment response can facilitate tailored therapies. Moreover, exploring combination therapies that target multiple immunological pathways simultaneously or sequentially may enhance treatment outcomes. This approach could involve combining biologic agents, small molecules, and other immunomodulatory therapies to achieve synergistic effects and better disease control. Additionally, Single-cell sequencing, omics analysis, and bioinformatics can provide valuable insights into the immune profiles and molecular signatures associated with RA. By harnessing these tools, we can unlock the potential to uncover novel therapeutic targets and create interventions that are more precise in their approach. Advancements will improve patient outcomes and enhance our understanding of complex immunological processes in RA.

## 9 Conclusion

Various therapeutic targets have been explored to address the pathogenesis of RA, and only a few have led to improved recovery in patients. Still, none have provided full protection against the disease. Hence, multitarget therapy, which has the potential to provide comprehensive protection, is now critically needed for better treatment. Notably, methotrexate was the first treatment approved for RA in the nineties, marking a significant milestone. Although methotrexate remains the first-line treatment, a new class of precise biologics in the form of antibody therapy has emerged, targeting multiple pathways to reduce joint inflammation effectively. Moreover, the FDA-approved next generation of JAK inhibitors (upadacitinib, baricitinib, tofacitinib), IL-6 receptor antagonists (sarilumab, tocilizumab), and TNF blockers (golimumab, certolizumab pegol, adalimumab, infliximab, etanercept) present emerging options for RA treatment. The necessity for precision therapy and preventing adverse effects are still priorities. To ensure optimal patient care and quality of life, study of disease pathways at the immuno-molecular level will uncover more specific therapy choices. In this review, the most prominent molecular therapeutic targets have been discussed, along with their role in pathology and regular physiology. This comprehensive review of these targets may be used when designing treatment strategies for any RA therapy.
